# ATM‐Dependent Recruitment of BRD7 is required for Transcriptional Repression and DNA Repair at DNA Breaks Flanking Transcriptional Active Regions

**DOI:** 10.1002/advs.202000157

**Published:** 2020-09-03

**Authors:** Kaishun Hu, Yu Li, Wenjing Wu, Limin Xie, Haiyan Yan, Yuexin Cai, Dong Chen, Qiongchao Jiang, Lehang Lin, Zhen Chen, Jian‐You Liao, Yin Zhang, H. Phillip Koeffler, Dong Yin, Erwei Song

**Affiliations:** ^1^ Guangdong Provincial Key Laboratory of Malignant Tumor Epigenetics and Gene Regulation Medical Research Center Sun Yat‐Sen Memorial Hospital Sun Yat‐Sen University Guangzhou 510120 China; ^2^ Department of Breast Oncology Sun Yat‐Sen Memorial Hospital Sun Yat‐Sen University Guangzhou 510120 China; ^3^ Department of Ultrasound Sun Yat‐Sen Memorial Hospital Sun Yat‐Sen University Guangzhou 510120 China; ^4^ Division of Hematology/Oncology Cedars‐Sinai Medical Center University of California Los Angeles School of Medicine Los Angeles CA 90048 USA

**Keywords:** ATM, BRD7, NuRD, PRC2, transcriptional repression

## Abstract

Repair of DNA double‐strand breaks (DSBs) is essential for genome integrity, and is accompanied by transcriptional repression at the DSB regions. However, the mechanisms how DNA repair induces transcriptional inhibition remain elusive. Here, it is identified that BRD7 participates in DNA damage response (DDR) and is recruited to the damaged chromatin via ATM signaling. Mechanistically, BRD7 joins the polycomb repressive complex 2 (PRC2), the nucleosome remodeling and histone deacetylation (NuRD) complex at the damaged DNA and recruits E3 ubiquitin ligase RNF168 to the DSBs. Furthermore, ATM‐mediated BRD7 phosphorylation is required for recruitment of the PRC2 complex, NuRD complex, DSB sensor complex MRE11‐RAD50‐NBS1 (MRN), and RNF168 to the active transcription sites at DSBs, resulting in transcriptional repression and DNA repair. Moreover, BRD7 deficiency sensitizes cancer cells to PARP inhibition. Collectively, BRD7 is crucial for DNA repair and DDR‐mediated transcription repression, which may serve as a therapeutic target. The findings identify the missing link between DNA repair and transcription regulation that maintains genome integrity.

## Graphical abstract

Repair of DNA double‐strand breaks (DSBs) is essential for genome integrity, and is accompanied by transcriptional repression at the DSB regions. ATM‐mediated BRD7 phosphorylation at the Ser 263 site is required for recruitment of the PRC2 complex, NuRD complex, DSB sensor complex MRE11‐RAD50‐NBS1 (MRN) and RNF168 to the active transcription sites at DSBs, resulting in transcriptional repression and DNA repair.

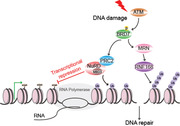

## 1 Introduction

DNA double‐strand break (DSB) is highly cytotoxic and deleterious lesions, causing gross chromosomal rearrangements, genomic instability, and tumorigenesis.^[^ ^1^ ^]^ In mammalian cells, such damages are mainly repaired through two pathways: non‐homologous end joining (NHEJ) and homologous recombination (HR).^[^ ^2^ ^]^ To avoid conflicts between ongoing transcription and repair activities, the DNA damage response (DDR) must coordinate the processes of repair signaling and local transcriptional capacity.^[^ ^3^ ^]^ Cells have evolved the ability of switching off transcription for DSB repair in ATM, DNA‐PK, and PARP1‐dependent manner which is a critical step for maintaining genome‐epigenome integrity.^[^ ^4^, ^5^, ^6^ ^]^ The mono‐ubiquitylation of H2A on K119 is one of the key determinants of transcriptional repression at sites of DNA damage.^[^ ^4^, ^7^, ^8^ ^]^ Several targets of ATM kinase and PARP1 have been implicated in catalyzing the formation of H2A‐K119 ubiquitination and subsequently led to transcription inhibition.^[^ ^1^, ^7^, ^9^, ^10^, ^11^, ^12^ ^]^ For instance, ATM kinase directly phosphorylates ENL and PBAF subunit BAF180 to promote monoubiquitylation of H2AK119, resulting in transcriptional repression and subsequently allowing DSB repair proteins to be recruited to DSBs site.^[^ ^8^, ^9^ ^]^ However, depletion of these factors did not completely alleviate DSB‐induced transcriptional repression, suggesting that additional yet unidentified factors or complexes are involved in the process.

Mammalian SWI/SNF (mSWI/SNF) ATP‐dependent chromatin remodeling complexes are evolutionarily conserved multi‐subunit molecular complexes: canonical BRG1/BRM‐associated factor (BAF), polybromo‐associated BAF complexes (PBAF) and non‐canonical BAFs (ncBAFs).^[^ ^13^, ^14^, ^15^ ^]^ Each complex has defined distinct components, and loss or mutation in specific subunits results in genome instability and carcinogenesis in a tissue‐specific manner.^[^ ^15^, ^16^, ^17^ ^]^ Specifically, PBAF complex, containing BAF180, BRD7 and ARID2 subunits, has important roles in regulating DNA accessibility for transcription, maintenance of chromatin architecture.^[^ ^18^, ^19^ ^]^ In addition to well‐established roles, PBAF complex has other important roles in DDR as transcriptional regulators and in both of HR and NHEJ repair^[^ ^8^, ^20^, ^21^, ^22^ ^]^ . Recently, two specific subunits of PBAF complex (ARID2 and PBAF) have been disclosed that are required for DSBs‐induced transcriptional repression.^[^ ^1^, ^8^ ^]^ However, how PBAF complex controls H2AK119Ub for transcriptional repression and how ATM kinase is involved in the process remain unclear.

In this study, we show that the BRD7, another specific subunit of PBAF complex, promotes transcriptional silencing and HR contributing to cell survival against DNA damage‐inducing agents. Moreover, our findings establish BRD7 as a newly identified modulator that orchestrates DSB‐induced transcriptional repression and DSBs repair signaling in transcriptionally active regions of chromatin, as well as highlight the important role of PBAF complex in maintaining genomic integrity.

## 2 Results

### 2.1 ATM, DNA‐PKs, and PARP1 Contributes to Transcriptional Repression at DNA DSBs

Transcription capacity is transiently and rapidly repressed in response to DNA damage, and the activity of DNA damage signaling enzymes ATM, DNA‐PK, and PARP1 have been implicated in DSB‐induced transcriptional repression.^[^ ^1^, ^4^, ^7^, ^9^, ^10^, ^23^, ^24^, ^25^ ^]^ Considering the notion that DNA damage inhibits transcription locally due to the stalling of RNA polymerase II (RNAP II), we monitored the status of actively RNAP II using indicated antibodies at the laser‐induced damage sites. As expected, phosphorylation of RNAP II on Ser 2, Ser 5, and Ser 7 were completely impaired at damage sites, suggesting that both transcriptional initiation (including pause release) and elongation are repressed upon either laser‐induced DSBs or Fok1‐induced DSBs (Figure S1A,B, Supporting Information).

To systematically investigate that the roles of DNA damage signaling enzymes in DSB‐induced transcriptional repression, U2OS‐263 reporter cells (kindly provided by Prof. Roger Greenberg^[^ ^26^ ^]^) were employed to evaluate the expression of CFP‐SKL reporter gene upon treatment with inhibitor of ATM, ATR, DNA‐PK, PARP, and PARG (Figure S1C, Supporting Information). Treatment with inhibitors (ATMi, DNA‐PKi or PARPi) led to transcription of MS2 gene and formation of YFP‐MS2 signal persistently at Fok1‐induced DSBs (Figure S1D–F, Supporting Information), suggesting that transcriptional repression in response to DNA damage is dependent on activity of ATM, DNA‐PK, and PARP.

Previous work concluded that ATM‐dependent H2A‐K119 ubiquitination was required for DSB‐induced transcriptional repression.^[^ ^4^, ^27^ ^]^ We tested whether DNA‐PK and PARP‐mediated transcriptional repression depends on ubiquitination of H2A‐K119 in response to DSBs. As expected, targeting ATM, DNA‐PK, and PARP with indicated inhibitors significantly decreased the H2A‐K119Ub accumulation and restored YFP‐MS2 signal formation at Fok1‐induced DSB sites (Figure S1G,H, Supporting Information). These data suggest that H2AK119 ubiquitination is required for ATM, DNA‐PK and PARP‐mediated transcriptional repression.

### 2.2 BRD7, a Subunit of PBAF Complex, Contributes to Transcriptional Repression at DNA Double‐Strand Breaks

**Figure 1:**
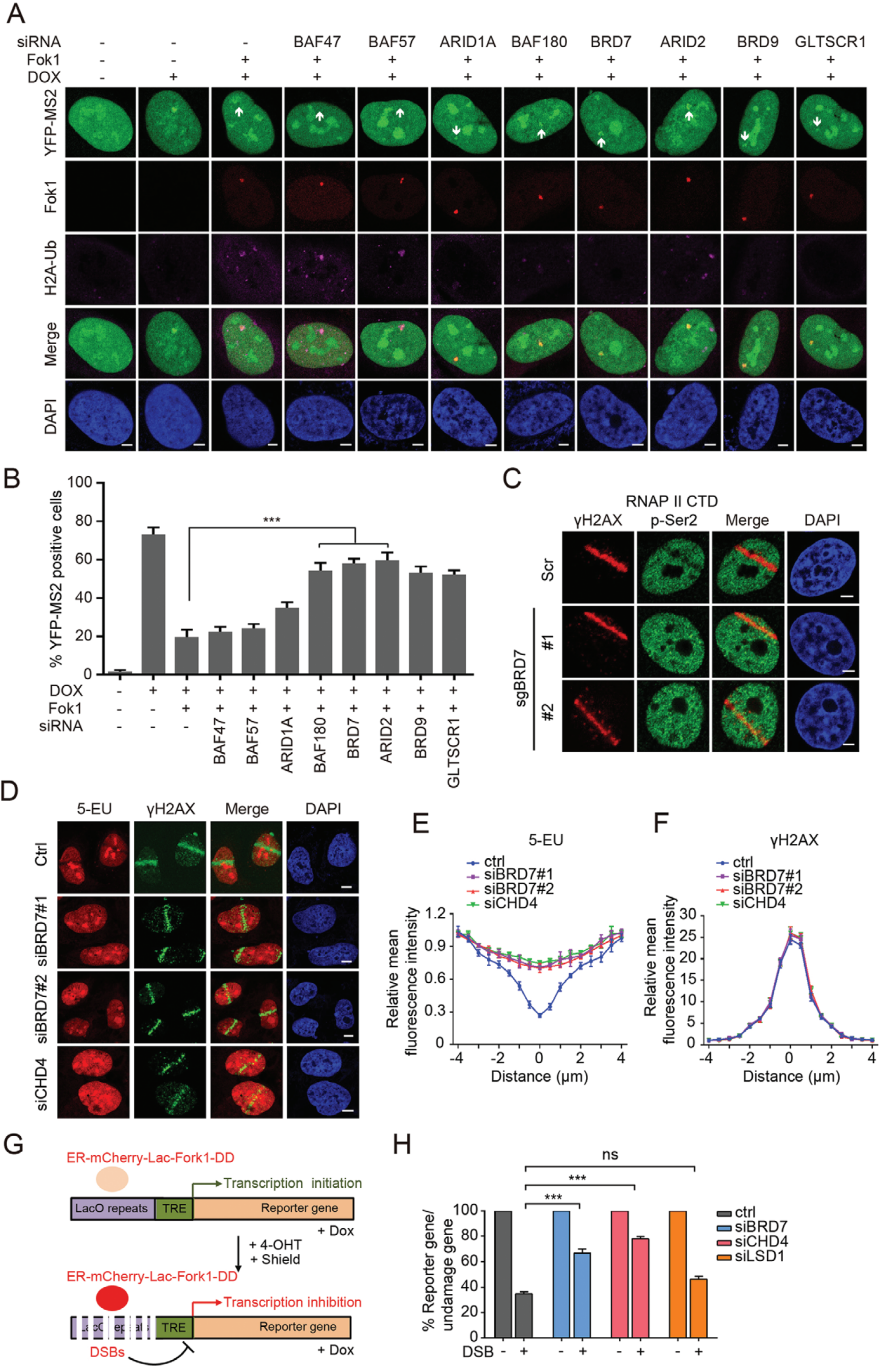
PBAF and ncBAF complex is required for transcriptional silencing induced by DNA DSBs. A) Transcriptional activity was measured in U2OS‐263 reporter cells transfected with small interfering RNA (siRNA) targeting either BAF47, BAF57, ARID1A, BAF180, BRD7, ARID2, BRD9, or GLTSCR1 upon induction of DSBs. Scale bars, 2 µm. B) Quantification of ongoing transcription in U2OS‐263 reporter cells treated with indicated siRNAs from experiments in panel (A). >100 cells were analyzed in each group. Data are presented as the mean ± SEM. C) DSBs‐induced exclusion of RNAP II Ser 2 from sites of laser damage was restored in BRD7‐depleted cells. Representative immunofluorescence images of U2OS cells treated with BRD7 sgRNA following by UV laser are shown. Scale bars, 2 µm. D) Depletion of BRD7 restored local inhibition of RNA synthesis visualized by 5‐EU incorporation. Cells treated with control or BRD7 siRNAs were subjected to 5‐EU label assay following laser damage and analyzed by immunofluorescence. E,F) Quantification of 5‐EU and *γ*H2AX fluorescence intensity from panel (D) using Image J software (NIH). Values were normalized to undamaged regions. Error bars indicate SEM; *n* > 10. G) Schematic of U2OS‐265 DSB reporter cells. Doxycycline induces transcription of the reporter gene, allowing be measured by quantitative PCR (qPCR) with indicated primers. Fok1‐mediated DSBs can be introduced by stabilizing ER‐mCherry‐Lac1‐Fok1‐DD with addition of Shield 1 and 4‐OHT. H) BRD7 and CHD4, but not LSD1, promote transcriptional repression at DSBs. The U2OS‐265 cells transfected with indicated siRNAs following by DSBs induction were subjected to qPCR analysis with indicated primers. Error bars indicate SEM. A pool of siCHD4#1 and siCHD4#2 was used for CHD4 knockdown treatments. n.s., not significant; **P < 0.01, ***P < 0.001, Student's *t*‐test.

Recent studies identified a role for PBAF complex in silencing transcription by promoting histone H2AK119 monoubiquitylation in response to DNA damage.^[^ ^1^, ^27^ ^]^ We tested whether BAF, PBAF, and ncBAF complexes are involved in the process. Consistent with previous findings, depletion of BAF180 and ARID2 resulted in a failure to silence transcription in Fok1‐positive cells (**Figure** **1**A,B and Figure S2A, Supporting Information). Strikingly, we also observed that depletion of BRD7 (a specific subunit of PBAF complex) and BRD9, GLTSCR1 (two specific subunits of ncBAF complex) also restored transcription activity and repressed H2AK119 monoubiquitylation after DNA damage (Figure 1A,B). Indeed, accumulation of the active form of RNA polymerase II (RNAP II CTD pSer 2) was decreased at break sites in control cells, but was significantly restored in BRD7‐depleted cells (Figure 1C and Figure S1B, Supporting Information).

To further confirm these results, we monitored nascent RNA transcript production using 5‐ethynyl uridine (5‐EU) labeling after UV‐laser induced DNA damage. Nascent RNA production was significantly rescued at sites of DNA damage in BRD7‐depleted cells (Figure 1D–F), similar with the results of depletion of CHD4, a known component of transcription repressed complex NuRD.^[^ ^28^ ^]^ Using another DSB reporter cells (U2OS‐265) developed by the Greenberg laboratory,^[^ ^26^ ^]^ we examined the status of transcriptional repression at a defined DSBs locus (Figure 1G). We observed that the depletion of BRD7 or CHD4 resulted in a defective transcriptional repression after DSBs induction (Figure 1H). In contrast, depletion of BHC complex member LSD1 has no effect on this pathway (Figure 1H). Taken together, these data demonstrate that the PBAF specific subunit BRD7 is important for maintaining DSBs‐induced transcriptional repression, and implied that a novel alternative pathway for PBAF complex in mediating transcriptional repression was identified.

### 2.3 BRD7 is Preferentially Recruited to DSBs nearby Transcriptionally Active Regions of Chromatin in ATM and PARP Activity Dependence Manner

**Figure 2:**
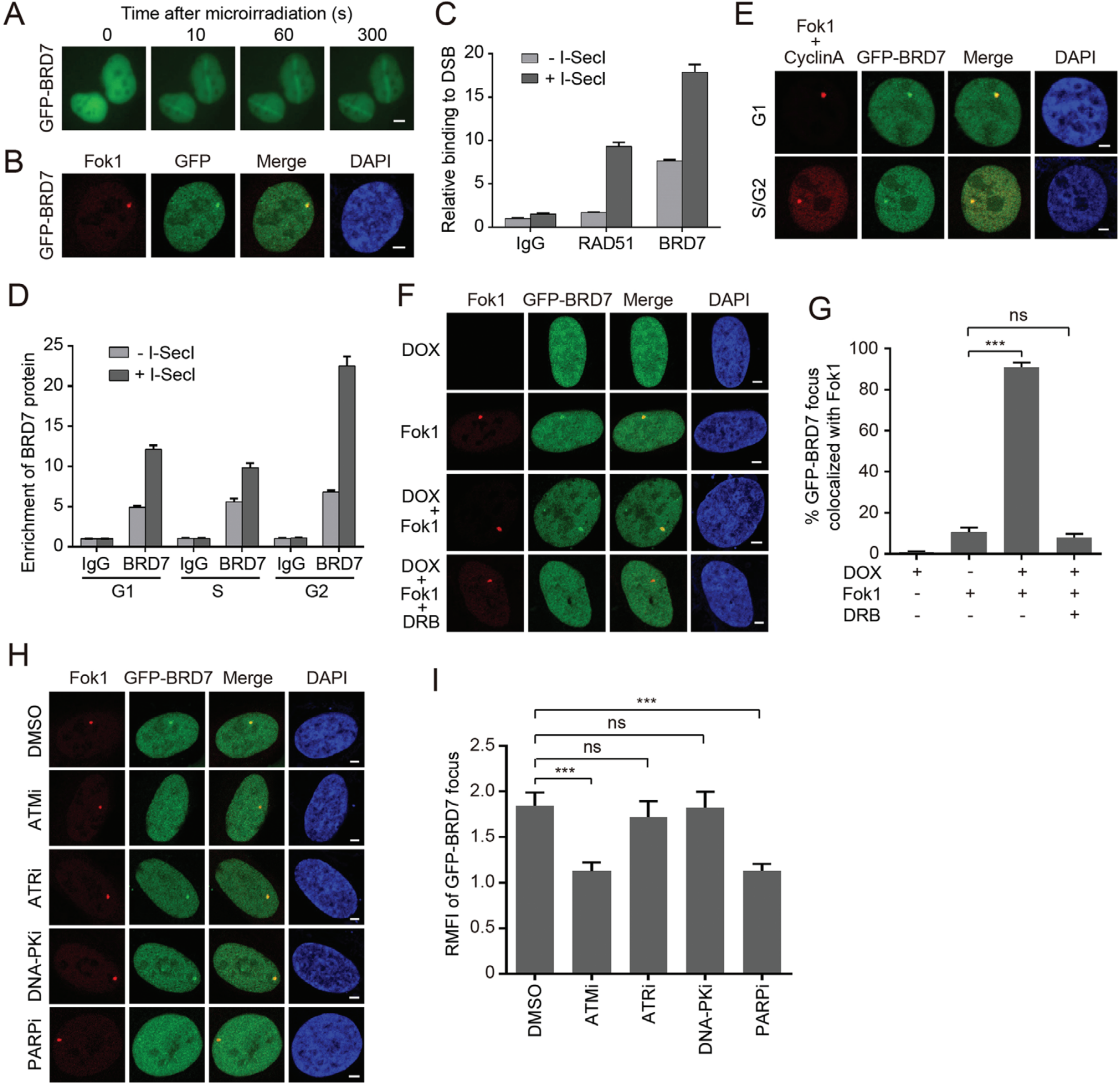
ATM‐dependent recruitment of BRD7 to DSBs nearby transcriptionally active regions. A) Recruitment of GFP‐BRD7 in laser damage. Representative images after UV laser damage are shown. B) BRD7 recruitment to Fok1‐induced DSBs. GFP‐BRD7 was transfected into U2OS‐265 DSB reporter cells, and cells were damaged by inducing site‐specific DSBs after 24 h transfection. Representative images after DNA damage are shown. C) BRD7 recruitment to I‐SecI‐induced DSBs analyzed by chromatin immunoprecipitation (ChIP) assay. Eight hours after I‐SecI transfection, ChIP assay was performed to detect the enrichment of BRD7 relative to the IgG control. D) The recruitment of BRD7 to DSBs increased throughout the cell cycle. HeLa DR‐GFP cells were treated with double‐thymidine to achieve cells at G1‐S boundary and then left unreleased (G1 phase) or released into the thymidine‐free medium for 3 h (S phase) or 7 h (G2 phase). The cells harvested at indicated phases were subjected to ChIP assay according to the Experimental Procedures. E) The recruitment of BRD7 to DNA damage was not restricted to S/G2 cells. Cyclin A is a S/G2 marker. GFP‐BRD7 was transfected into U2OS‐265 DSB reporter cells, and cells were introduced site‐specific DSBs. Representative images after DNA damage are shown. F) GFP‐BRD7 is preferentially recruited to DSBs induced upstream of active gene. U2OS‐265 DSB reporter cells were first treated with 1 µg mL^−1^ doxycycline for 2 h to induce nascent transcription of the reporter gene, and then cells were induced site‐specific DSBs after 24 h transfection of GFP‐BRD7. Representative images after DNA damage are shown. G) Quantification of GFP‐BRD7 focus colocalized with Fok1 of panel (F). Data are represented as mean ± SEM. H) ATM and PARP1, but neither ATR nor DNA‐PK is required for the recruitment of BRD7 to DSBs. U2OS‐265 DSB reporter cells were transfected with GFP‐BRD7 for 24 h, then ATM inhibitor (Ku55933, 10 × 10^−6^ m), ATR inhibitor (VE‐821, 10 × 10^−6^ m), DNA‐PK inhibitor (NU7441, 5 × 10^−6^ m), PARP inhibitor (Olaparib, 10 × 10^−6^ m) were added for additional 4 h, followed by introduced site‐specific DSBs. Representative images after DNA damage are shown in (H). All the scale bars are 2 µm. I) Quantification of GFP‐BRD7 relative mean fluorescence intensity (RMFI) from H) calculated from 50 cells using Image J software (NIH) and three independent experiments. Error bars indicate SEM. n.s., not significant; **P < 0.01, ***P < 0.001, Student's *t*‐test.

To gain insights into whether BRD7 proteins directly contribute to the DDR, we monitored BRD7 subcellular localization upon DSBs. Chromatin was extracted to investigate whether BRD7 modulates DSB repair in the context of chromatin. A portion of the BRD7 protein remained in the chromatin‐associated fraction (Figure S3A,B, Supporting Information). Indeed, GFP‐BRD7 could rapidly recruit to laser‐microirradiated sites as early as 10 s with peak intensity at about 1 min (**Figure** **2**A). To further corroborate the recruitment of BRD7 to DSBs, we investigated the accumulation of BRD7 at Fok1‐induced DSB using the reporter U2OS‐265 cells. As expected, accumulation of GFP‐BRD7 was observed at Fok1‐positive regions (Figure 2B). To support this finding, we expressed the I‐SceI endonuclease to introduce DSBs in HeLa cells carrying an integrated DR‐GFP reporter^[^ ^29^ ^]^ (Figure S3C, Supporting Information). A chromatin immunoprecipitation (ChIP) assay showed that BRD7 at the I‐SceI break site increased after I‐SceI‐induced DSB comparable with the results of using anti‐RAD51 antibodies (Figure 2C and Figure S3D,E, Supporting Information). Interestingly, we found that BRD7 was enriched at DNA lesion was not restricted to S/G2 phase, as established by targeting BRD7 ChIP in G1 phase cells and co‐staining with the S/G2 marker Cyclin A (Figure 2D,E and Figure S3F,G, Supporting Information). Moreover, remarkable accumulation of GFP‐BRD7 colocalized with Fok1 foci was observed after pretreatment with Dox following Fok1 induction in U2OS‐265 reporter cells (Figure 2F,G) and this process was mainly governed by ATM kinase and PARP1 (Figure 2H,I). Taken together, these results suggest that BRD7 accumulates at DSBs nearby transcriptionally active regions in ATM and PARP activity dependence manner.

### 2.4 BRD7 Interacts with the PcG Complex PRC2 and the Chromatin Remodeling Complex NuRD, and Recruits PRC2 and NuRD Complexes at Transcription Sites in Response to DSBs

**Figure 3:**
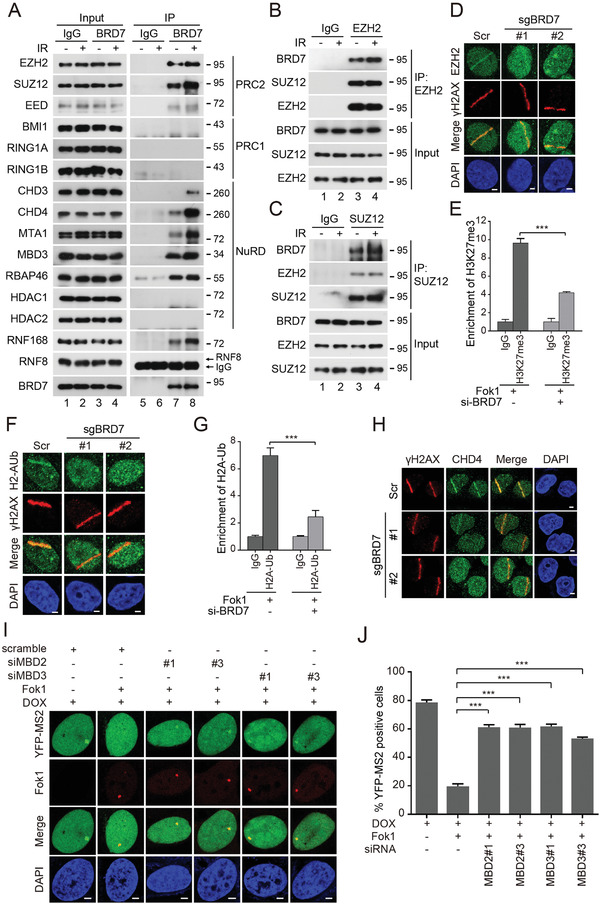
BRD7 associates with PRC2, NuRD complexes and recruits these factors to damaged chromatin. A–C) BRD7 interacts with PRC2 and NuRD complexes, but not with PRC1 complex. HeLa cells were treated with IR (5 Gy), followed by IP using either anti‐IgG, anti‐BRD7, anti‐EZH2 or anti‐SUZ12 antibodies, and analyzed by Western blot. D,F) Recruitment of EZH2 and H2A‐Ub to laser damage requires BRD7. Control and BRD7‐depleted cells were subjected to UV laser, and endogenous EZH2 and H2A‐Ub accumulation was analyzed by immunofluorescence. E,G) ChIP‐PCR was performed in U2OS‐265 reporter cells with and without Fok1‐induced DSBs using either anti‐IgG or H3K27me3 or H2AK119Ub antibodies. Error bars indicate SEM from three independent experiments. H) BRD7 recruits CHD4 to the DSB site. Control and BRD7‐depleted cells were subjected to UV laser, and endogenous CHD4 accumulation was analyzed by immunofluorescence. All the scale bars are 2 µm. I,J) Transcriptional activity was measured in U2OS‐263 reporter cells transfected with small interfering RNA (siRNA) targeting either MBD2 or MBD3 upon induction of DSBs. Scale bars, 2 µm. J) Quantification of ongoing transcription in U2OS‐263 reporter cells treated with indicated siRNAs from experiments in panel (I). >50 cells were analyzed in each group. Data are presented as the mean ± SEM. n.s., not significant; **P < 0.01, ***P < 0.001, Student's *t*‐test.

To investigate the mechanism of BRD7 involved in DSBs‐induced transcriptional repression, we established a HeLa cell line stably expressing SFB‐BRD7 and identified its interacting proteins by tandem affinity purification and mass spectrometry. EZH2 and CHD3 were identified to interact with BRD7 (Table S1, Supporting Information). PRC2 is a conserved complex that contains EZH2, SUZ12, EED, and RbAp46/48.^[^ ^30^, ^31^ ^]^ EZH2 promotes the trimethylation of histone H3 lysine 27 (H3K27me3) facilitating recruitment of the ubiquitin ligase PRC1.^[^ ^32^ ^]^ PRC1 is a multiple subunits complex and catalyzes the ubiquitylation of histone H2AK119, leading to transcription silencing through the induction of chromatin compaction.^[^ ^33^ ^]^ Mammalian NuRD complexes can act as transcriptional corepressors to promote transcriptional silencing.^[^ ^34^, ^35^ ^]^ As shown in **Figure** **3**A–C, a complex containing either BRD7/PRC2 or BRD7/NuRD, but not BRD7/PRC1, was clearly detected. Most importantly, the interaction of BRD7 with PRC2 and NuRD was greatly enhanced following exposure to IR (irradiation) (Figure 3A–C). In addition, this interaction of BRD7 with PRC2 and NuRD appears to be direct, as recombinant GST‐BRD7 binds PRC2 and NuRD in vitro (Figure S4A, Supporting Information). Collectively, these results indicate that BRD7 may physically interact with PRC2 and NuRD in vitro and in vivo.

To further explore the functional relationship between BRD7, PRC2, and NuRD complexes after DNA damage, we investigated their recruitment to DSBs sites with depletion of each subunits. Depletion of either EZH2 or SUZ12 did not affect GFP‐BRD7 accumulation at damage sites (Figure S5A–D, Supporting Information), however, depletion of BRD7 dramatically reduced EZH2 and H2AK119Ub accumulation at damage sites (Figure 3D–F). Furthermore, we performed ChIP‐PCR with indicated primers targeting a region downstream of the DSBs, and the levels of both H3K27me3 and H2AK119Ub accumulation at the sites of Fok1‐induced DSBs were significantly decreased after depletion of BRD7 (Figure 3E–G), indicating that BRD7 greatly contribute to the trimethylation of H3K27 and monoubiquitylation of H2AK119. Meanwhile, depletion of BRD7 remarkedly reduced CHD4 accumulation at damage sites (Figure 3H). Since the MBD3‐containing NuRD complex in mammals can be targeted to methylated DNA by MBD2,^[^ ^36^ ^]^ we assumed that MBD2 and MBD3 might be involved in DSB‐induced transcriptional inhibition. Indeed, we observed that the depletion of MBD2 or MBD3 resulted in a defective transcriptional repression after DSBs induction (Figure 3I,J and Figure S5E,F, Supporting Information), suggesting that DNA methylation at DSB site also contributes to DSB‐induced transcriptional inhibition. Together, these data suggested that BRD7 is required for the recruitment of transcriptional repressor complex PRCs and NuRD to DSBs sites.

### 2.5 BRD7 Interacts with RNF168 and Induces its Accumulation at DSBs

**Figure 4:**
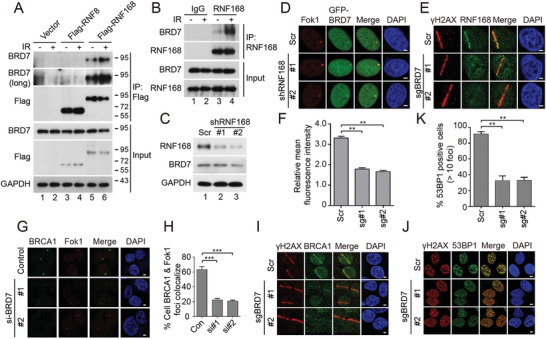
BRD7 recruits RNF168 to the DSB site. A,B) BRD7 interacts with RNF168 but not with RNF8. HeLa cells transfected with Flag‐RNF8 or Flag‐RNF168 were treated with IR (5 Gy), followed by IP using anti‐Flag, anti‐IgG or RNF168 antibodies, and Western blot was performed with indicated antibodies. C) U2OS‐265 cells were infected with RNF168 specific shRNAs and cultured in DMEM medium containing blasticidin for 72 h. Cells were lysed with RIPA buffer, and analyzed using Western blot. D) RNF168 depletion did not affect the accumulation of GFP‐BRD7 to Fok1‐induced DSBs. The U2OS‐265 cells depleted RNF168 were transfected with GFP‐BRD7 for 24 h and cells were subjected to introduce site‐specific DSBs. Representative images after DNA damage are shown. E) Recruitment of RNF168 to laser damage requires BRD7. Control and BRD7‐depleted cells were subjected to UV laser, and endogenous RNF168 accumulation was analyzed by immunofluorescence. F) Quantification of RNF168 relative mean fluorescence intensity (RMFI) from E) calculated from 50 cells using Image J software (NIH) and three independent experiments. Error bars indicate SEM. G,H) U2OS‐265 cells were transfected with either scrambled or BRD7 siRNAs for 48 h, and cells were induced site‐specific DSBs, followed by analysis of immunofluorescence. Representive images after DNA damage are shown. Scale bars, 2 µm. H) Quantification of % BRCA1 colocalized with Fok1 in panel (G). Error bars indicate SEM; *n* = 3. I) BRD7 recruits BRCA1 to the DSB site. Control and BRD7‐depleted cells were subjected to UV laser, and endogenous BRCA1 was analyzed by immunofluorescence. All the scale bars are 2 µm. J,K) BRD7 depletion reduces 53BP1 foci formation. BRD7‐depleted HeLa cells were treated with IR (5 Gy) and allowed to recover for 30 min before fixing and processed for 53BP1 and *γ*H2AX immunofluorescence. Quantification results are the average of three independent experiments and are presented as mean ± SEM. More than 100 cells were counted in each experiment. n.s., not significant; **P < 0.01, ***P < 0.001, Student's *t*‐test.

During the DDR, ubiquitin signaling plays a vital role in orchestrating the recruitment of DNA repair proteins, such as BRCA1 and 53BP1.^[^ ^37^, ^38^, ^39^, ^40^ ^]^ RNF8 and RNF168 are two important ubiquitin E3 ligases involving in these ubiquitylation events nears DSBs.^[^ ^39^, ^41^ ^]^ To test whether the accumulation of BRD7 at DSB sites was dependent on physical interaction with RNF8 or RNF168, we performed co‐IP experiments using transient transfection either Flag‐RNF8 or Flag‐RNF168. As shown in Figure 3A, a complex containing BRD7 and RNF168 was clearly detected. Moreover, endogenous Co‐IP also suggested that both BRD7 and RNF168 existed in the immunoprecipitated complex, and the interaction of BRD7 with RNF168 was greatly enhanced by exposure to IR (**Figure** **4**A,B). We next tested the possibility that there could be a direct protein–protein interaction between BRD7 and RNF168. GST pull‐down assays using recombinant GST‐tagged BRD7 purified from *Escherichia coli* showed that BRD7 strongly interacts with RNF168 (Figure S4A, Supporting Information). Furthermore, depletion of RNF168 did not affect accumulation of GFP‐BRD7 at damage sites (Figure 4C,D). To understand the role of BRD7 in RNF8‐RNF168 DNA damage response pathway, we depleted endogenous BRD7 and probed various DNA damage response proteins. BRD7 did not affect the recruitment of Ku70, Ku80 as well as RNF8 to DSBs (Figure S5G–I, Supporting Information). Strikingly, RNF168 and its downstream effector BRCA1, 53BP1 accumulation at damage sites were dramatically reduced in BRD7 depleted cells (Figure 4E–K), suggesting that BRD7 plays a role in upstream of RNF168. These data strongly suggested that BRD7 is required for the recruitment of ubiquitin E3 ligase RNF168 and its downstream effector BRCA1 and 53BP1 to DNA damage sites.

### 2.6 BRD7 is Essential for Cellular Response to DSB Damage, and BRD7 Depletion Inhibits Both the HR and NHEJ Efficiencies

To investigate the function of BRD7 protein in DSB repair, we examined the effect on cell viability following treatment with CPT. Remarkably, BRD7 depletion rendered cells more sensitive toward CPT (Figure S6A, Supporting Information). We further analyzed *γ*H2AX foci formation in control or BRD7‐depleted cells to confirm that whether BRD7 has a role in DSB repair. As shown in Figure S6B–F (Supporting Information), BRD7 depletion cells showed the persistent *γ*H2AX‐positive at the late repair stage and elevated levels of spontaneous *γ*H2AX foci compared with control cells. These results suggest that BRD7 is required for DSB repair timely.

DSBs are repaired by two major pathways: HR or NHEJ. We used DR‐GFP or EJ5‐GFP reporter cells to detect which pathway BRD7 affecting on the repair of DSB (Figure S6G,I, Supporting Information).^[^ ^42^, ^43^ ^]^ As shown in Figure S6H (Supporting Information), depletion of BRD7 impaired HR repair efficiency to a level like that achieved by depleting the key HR factor RAD51 (Figure S7A,B, Supporting Information). Moreover, BRD7 depletion greatly inhibited efficiency of NHEJ repair (Figures S6I,J and S7C,D, Supporting Information). Next, we examined whether BRD7 depletion affects DSB repair via single‐strand annealing (SSA), which usually arises from the annealing of complementary single strands formed after resection at a DSB, resulting in a deletion of repeat sequences. We found that BRD7 depletion decreased the efficiency of SSA, whereas depletion of RAD51 increased the efficiency of SSA as a positive control (Figures S6K,L and S7E,F, Supporting Information). To determine if BRD7 promotes HR repair in a manner independent or dependent on the chromatin remodeling activity of the PBAF complex, we depleted endogenous BRD7 alone or in combination with BRG1 and BAF180, two core subunits of PBAF complex, to investigate its effect toward HR repair. As shown in Figure S6O,P (Supporting Information), double depletion of BRG1 or BAF180 combined with BRD7‐depleted significantly reduce HR repair efficiency, suggesting that BRD7 promotes HR repair independent on the chromatin remodeling activity of the PBAF complex. Importantly, suppression of BRD7 led to an increase of S phase population, which should be predicted to be repaired by HR, indicating that the observed phenotypes in BRD7‐depleted cells were not caused by cell‐cycle fluctuation (Figure S7G, Supporting Information).

Deficiency in the HR pathway renders cells more sensitive toward CPT, ionizing radiation (IR), and PARP inhibitors.^[^ ^44^ ^]^ Since BRD7 depletion suppresses HR, we further investigated the role of BRD7 in response to PARP inhibition. We found that depletion of the BRD7 rendered both HeLa and MDA‐MB‐231 cells sensitive to PARP inhibitors (Figure S6M,N, Supporting Information). These results suggest that, in addition to its role in NHEJ repair, BRD7 also functions during a step common toward homologous‐repair pathways to promote both HR and SSA, unlike downstream effector RAD51 to promotes HR but suppresses SSA.

### 2.7 BRD7 Interacts with MRN Complex and is required for Recruitment of the MRN Complex at DSBs

**Figure 5:**
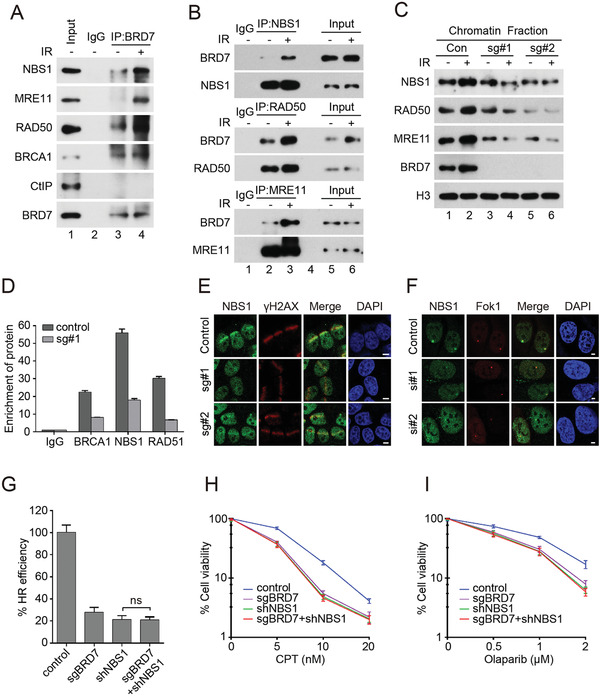
BRD7 binds and recruits MRN complex to DSBs sites. A,B) BRD7 interacts with MRN complex and BRCA1. HeLa cells were treated first with 5 Gy of IR and lysed with RIPA buffer after 1 h, and lysates were subjected to immunoprecipitation, and analyzed by Western blot. C) BRD7 depletion suppressed the recruitment of MRN complex to damaged chromatin. HeLa cells depleted of BRD7 were treated with IR (5 Gy) and allowed to release for 1 h. Chromatin binding proteins were isolated according to the Supplemental Experimental Procedures followed by Western blot analysis. D) BRD7 depletion reduces recruitment of BRCA1 and NBS1 to I‐SecI‐induced DSBs. Eight hours after I‐SecI transfection, ChIP assay was performed to detect the enrichment of BRCA1, NBS1, RAD51 relative to the IgG control. Quantification results are the average of three independent experiments and are shown as mean ± SEM. E) BRD7 depletion impairs recruitment of NBS1 to laser damage. Control and BRD7‐depleted cells were subjected to UV laser, and endogenous NBS1 accumulation was analyzed by immunofluorescence. Scale bars, 5 µm. F) BRD7 knockdown suppresses Fok1‐induced NBS1 foci formation. Control and BRD7 knockdown U2OS‐265 cells were induced site‐specific DSBs and processed for NBS1 immunofluorescence. Representative NBS1 foci are shown. Scale bars, 2 µm. G) Control and BRD7 knockout HeLa DR‐GFP cells stably expressing NBS1 shRNA were electroporated with I‐SceI plasmid. 48 h after transfection, cells were harvested and performed for GFP expression by flow cytometry analysis (FACS). Quantification results are the average of three independent experiments and are shown as mean ± SEM. H,I) The sensitivity of control cells and BRD7 knockout HeLa cells with stably expressing NBS1 shRNA to CPT or Olaparib were assessed using colony formation assay. The cell lines were treated with indicated doses of CPT or Olaparib, and cell survival were measured. Results shown are averages of three independent experiments.

MRN complex is required for ATM kinase recruitment and activation, and the resultant formation and expansion of *γ*H2AX foci at DSBs.^[^ ^45^ ^]^ We tested whether there is a functional link between BRD7 and MRN complex in response to DSBs. As shown **Figure** **5**A, endogenous BRD7 could co‐immunoprecipitate with NBS1, MRE11, RAD50 as well as BRCA1 (a BRD7‐binding protein has been previously reported^[^ ^46^ ^]^), but not with CtIP, and these interactions are dramatically enhanced following DSB induction. A reciprocal IP also showed that an increase level of interaction between BRD7 and MRN complex components upon DSB induction (Figure 5B). We next tested the possibility that there could be a direct protein‐protein interaction between BRD7 and MRN complex. GST pull‐down assays using recombinant GST‐tagged BRD7 purified from *E. coli* showed that BRD7 strongly interacts with MRN complex (Figure S4A, Supporting Information). BRD7 have a conserved bromodomain (BD, 128–238 amino acids) which specifically recognizes acetylated lysine on histones and the amino‐terminal part of BRD7 (residues 1–128) responsible for interaction with p53^[^ ^47^ ^]^ (Figure S4B, Supporting Information). Deletion of individual regions of BRD7 demonstrated that the BRD7 and BRD7 (ΔBD, a mutant lacking the bromodomain) bound equally well to MRN complex, but depletion of 1–128 domain significantly decreased the binding ability to MRN complex (Figure S4C, Supporting Information), indicating that the bromodomain of BRD7 is not required for this interaction. To examine whether BRD7 is required for recruitment of the MRN complex to damaged DNA, we performed chromatin fraction assay and found that depletion of BRD7 reduced the binding of MRN complex to chromatin after exposure IR (Figure 5C). Moreover, BRD7 depletion decreased NBS1 and BRCA1 recruitment to I‐SceI‐induced DSBs (Figure 5D). Since NBS1 is a key component of the MRN complex, and acts as a scaffold protein mediated the interaction of MRE11 and ATM,^[^ ^48^ ^]^ we investigated the effect of BRD7 depletion on NBS1 accumulation to DSBs. As shown in Figure 5E,F, NBS1 accumulation at both laser‐induced damage and Fok1‐induced DSB sites were dramatically reduced in BRD7‐depleted cells. To further confirm that BRD7 regulated HR repair through the direct interaction of MRN complex, we introduced shRNA targeting endogenous NBS1 into BRD7‐depleted cells. As shown in Figure 5G–I, NBS1‐deficiency compromised the HR and sensitized cells to CPT and Olaparib treatment. Furthermore, loss of BRD7 had no further effect on HR efficiency and chemosensitivity in NBS1 deficient cells, suggesting that NBS1 was a downstream target of BRD7 (Figure 5G–I). Taken together, these results suggest that BRD7 regulates HR and DDR in a NBS1‐dependent manner.

### 2.8 BRD7 Promotes DNA‐End Resection and is required for a Proper G2/M DNA Damage Checkpoint

**Figure 6:**
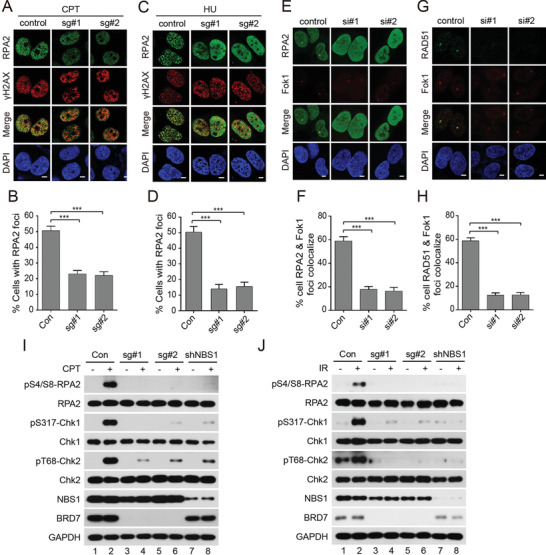
BRD7 depletion inhibits DNA‐end resection. A,B) BRD7 depletion impairs CPT‐induced RPA2 foci formation. Control and BRD7‐depleted HeLa cells were treated with CPT (1 × 10^−6^ m) for 1 h before fixing and subjected to immune staining analysis with indicated antibodies. Representative RPA2 foci are shown in (A). Quantification results are the average of three independent experiments and are showed as mean ± SEM in panel B. More than 100 cells are counted in each assay. C,D) BRD7 depletion inhibits HU‐induced RPA2 foci formation. Control and BRD7‐depleted HeLa cells were treated with HU (10 × 10^−3^ m) for 1 h before fixing and subjected to immune staining analysis with indicated antibodies. Representative RPA2 foci are shown in (C). Quantification results are the average of three independent experiments and are showed as mean ± SEM in panel (D). More than 100 cells are counted in each assay. E–H) BRD7 depletion impairs Fok1‐induced RPA2 and RAD51 foci formation. Control and BRD7‐depleted U2OS‐265 cells were induced site‐specific DSBs and processed for RPA2 and RAD51 immunofluorescence. Representative RPA2 and RAD51 foci are shown in (E,G). Quantification results are the average of three independent experiments and are showed as mean ± SEM in panel (F,H). More than 100 cells are counted in each assay. I,J) BRD7 depletion impairs RPA2, CHK1, and CHK2 phosphorylation following CPT or IR treatment. Control and BRD7 or NBS1‐depleted HeLa cells were harvested at 1 h after cells exposure to 1 × 10^−6^ m CPT or 5 Gy IR and cell lysates subjected to western blot analysis with indicated antibodies. Scale bars, 5 µm. n.s., not significant; **P < 0.01, ***P < 0.001, Student's *t*‐test.

MRN complex is essential for HR repair and the initiation of DSB processing via the endonuclease activity of MRE11.^[^ ^49^ ^]^ HR repair is initiated by nuclease‐mediated DNA end resection to generate single stranded DNA (ssDNA) that are initially protected by the replication protein A (RPA) complex and subsequently displaced by RAD51 recombinase.^[^ ^2^, ^50^ ^]^ Thus, failure of RPA2 and RAD51 accumulations at DSB sites are indicative of a defect in DNA‐end resection. Since BRD7 is critical for MRN complex recruitment to DSBs, we speculated that BRD7 depletion may also affect RPA2 and RAD51 foci formation upon DNA damage. As expected, RPA2 foci were dramatically reduced in BRD7‐depleted cells in response to CPT treatment (**Figure** **6**A,B). Moreover, both RPA2 and RAD51 foci formation were severely impaired in BRD7‐depleted U2OS‐265 cells upon Fok1‐induced DSBs (Figure 6E–H). Interestingly, BRD7 depletion also inhibited HU‐induced RPA2 focus formation, indicating that BRD7 depletion impairs RPA2 recruitment in response to DNA damage, as well as replication stress (Figure 6C,D).

MRN/ATM‐dependent formation of RPA‐coated ssDNAs intermediator is not only required for the HR repair but also for activation of ATR‐dependent signaling of DSBs.^[^ ^2^, ^51^, ^52^ ^]^ BRD7 depletion significantly attenuated both CPT‐ and IR‐induced CHK1 and RPA2 phosphorylation (Figure 6I,J). Depletion of BRD7 also dramatically decreased CHK2 phosphorylation after exposure to CPT and IR treatment (Figure 6I,J), suggesting that BRD7 is required for facilitating DNA‐end resection, ATR‐dependent signaling and activation of ATM/CHK2 signaling.

Next, we tested whether BRD7 deficiency impairs the cellular response to DNA damage by examining cell cycle distribution. As shown in Figure S8A,B (Supporting Information), depletion of BRD7 markedly reduced proportion of cells arrested at G2‐M phase. To further clarify these results, we performed phospho‐histone H3 staining to measure the proportion of mitotic cells after exposure to CPT in BRD7‐depleted cells.^[^ ^53^ ^]^ Depletion of BRD7 led to bypass the active G2‐M checkpoint and a significantly increased percentage of cells reentering mitosis (Figure S8C,D, Supporting Information), suggesting that BRD7 depletion impairs G2‐M checkpoint initiation and maintenance. Consistent with our previous finding, depletion of BRD7 inhibited mitotic entry and decreased the percentage of cells at mitotic phase comparing to normal conditions (Figure S8E, Supporting Information).^[^ ^53^ ^]^ However, BRD7 depletion resulted in a marked increase of mitotic cells after release from CPT treatment (Figure S8E,F, Supporting Information). Consistently, BRD7 depletion displayed a markedly earlier decline in the initiation and maintenance of CHK1 and CHK2 phosphorylation compared to control cells (Figure S8G,H, Supporting Information). Taken together, these data strongly suggested that BRD7 is required for DNA end resection activation and G2‐M checkpoint initiation and maintenance.

### 2.9 ATM Directly Phosphorylates BRD7 at Ser 263 Site

**Figure 7:**
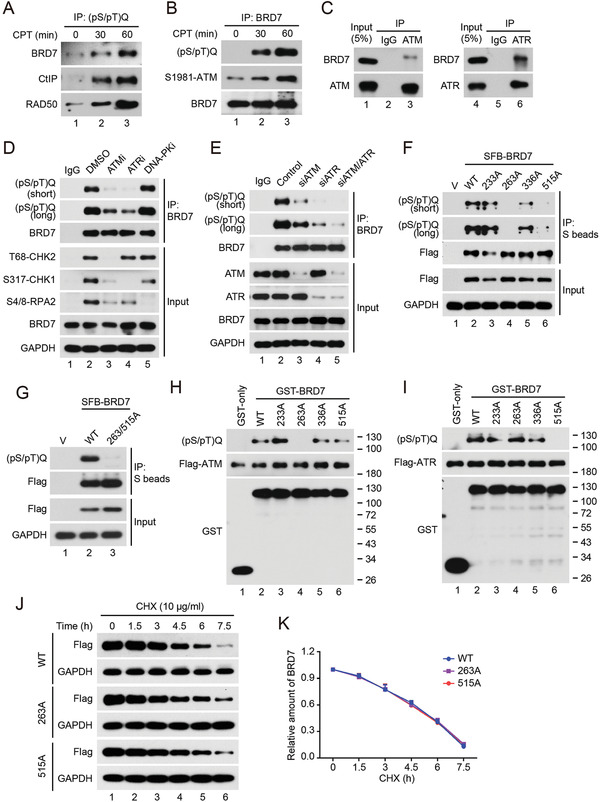
ATM and ATR directly phosphorylate BRD7 at Ser 263 and Thr 515 sites respectively. A,B) HeLa cells were treated with CPT (1 × 10^−6^ m) for different time intervals followed by lysing with RIPA buffer, and lysates were subjected to immunoprecipitation using either anti‐(pS/pT)Q or anti‐BRD7 antibodies and immunoblotted with the indicated antibodies. C) HeLa cells treated with CPT (1 × 10^−6^ m, 1 h) were lysed with RIPA buffer, and lysates were subjected to immunoprecipitation using either anti‐IgG, or ATM or ATR antibodies, and analyzed by Western blot. D) HeLa cells were treated first with DMSO, KU55933 (ATM inhibitor, 10 × 10^−6^ m), VE‐821 (ATR inhibitor, 10 × 10^−6^ m) or NU7441 (DNA‐PK inhibitor, 1 × 10^−6^ m) for 2 h followed by treatment with CPT for another 1 h, and the cells were lysed with RIPA buffer, and subjected to immunoprecipitation using either anti‐IgG, or BRD7 antibodies, and analyzed by Western blot with the indicated antibodies. E) HeLa cells were transfected with indicated siRNAs for 48 h followed by treatment with CPT for another 1 h, and then were lysed with RIPA buffer, and were subjected to immunoprecipitation using indicated antibodies. F) HeLa cells were transfected with BRD7 wild‐type and various BRD7‐mutant plasmids for 24 h followed by treatment with CPT for another 1 h, lysed with RIPA buffer, followed by immunoprecipitation and Western blot with indicated antibodies. G) HeLa cells were transfected with BRD7 wild‐type and BRD7 double mutant plasmids for 24 h followed by CPT treatment for another 1 h, and analyzed by Western blot. H) ATM directly phosphorylates BRD7 at Ser 263. An in vitro ATM assay was performed as described in Experimental Section. I) ATR directly phosphorylates BRD7 at Thr 515. An in vitro ATR assay was performed as described in Supplementary Material and Methods. J) HeLa cells were transfected with either BRD7 wild‐type, BRD7‐S263A or BRD7‐T515A for 24 h, followed by incubation with 10 µg mL^−1^ cycloheximide (CHX) for the indicated periods of time. Lysates were harvested and analyzed by Western blot. K) Quantification of BRD7 protein levels from panel (J), *n* = 3. Error bars indicate SEM. Relative amounts normalized to the BRD7 protein level at 0 h.

BRD7 recruitment to DSBs was dependent on ATM kinase activity, we speculated that BRD7 is a substrate of ATM. Indeed, a clear band of phosphorylated BRD7 was detected by immunoprecipitation using either anti‐phospho‐SQ/TQ antibody or anti‐BRD7 antibody upon CPT treatment (**Figure** **7**A,B). Moreover, an endogenous complex containing BRD7 and ATM, ATR were also detected (Figure 7C). Next, we detected the kinase(s) for BRD7 phosphorylation using selective inhibitors against ATM, ATR, and DNA‐PK. In contrast to inhibition of DNA‐PK did not affect BRD7 phosphorylation, the ATM inhibitor KU55033 and ATR inhibitor VE‐821 dramatically eliminated CPT‐induced BRD7 phosphorylation (Figure 7D). Furthermore, BRD7 phosphorylation almost completely inhibited by depletion of either endogenous ATM or ATR or combination upon treatment with CPT (Figure 7E), suggesting that BRD7 is a direct substrate of ATM and ATR. BRD7 possesses four SQ/TQ motifs (Figure S9A,B, Supporting Information), which are potential phosphorylation sites of ATM/ATR/DNA‐PK kinases .To define which SQ/TQ site is phosphorylated by ATM and ATR, four BRD7 single mutants were used and found that CTP‐induced BRD7 phosphorylation was greatly decreased in the BRD7‐S263A and BRD7‐T515A mutant, while mutation of BRD7 S233A or S336A had no effect (Figure 7F). Consistent with this result, the double mutant of BRD7‐S263A/T515A abrogated completely phosphorylation of BRD7 upon CPT treatment (Figure 7G). The phosphorylation of BRD7 at Ser 263 and Thr 515 sites in cells were further confirmed by mass spectrometry analysis (Figure S9C, Supporting Information). To further clarify the site(s) of BRD7 required for ATM/ATR‐mediated phosphorylation, we performed an in vitro ATM/ATR kinase assay and showed that ATM, ATR could phosphorylate wild‐type BRD7 at Ser 263 and Thr 515 sites, respectively (Figure 7H,I). The post‐translational modification, including poly(ADP‐riosyl)ation and ubiquitination, has been reported to modulate BRD7 stability.^[^ ^54^, ^55^ ^]^ We tested whether ATM and ATR contributed to BRD7 degradation. As shown in Figure 7J,K, half‐life of BRD7 S263A and T515A mutants have no significant difference compared with BRD7 wild‐type. Taken together, these results indicate that ATM is the major kinase responsible for BRD7 phosphorylation at Ser 263, and ATR kinase contributes to BRD7 phosphorylation at Thr 515 after DNA damage.

### 2.10 ATM‐Dependent Phosphorylation of BRD7 at Ser 263 is required for DSB‐Induced Transcriptional Repression, HR Repair and Activation of ATM/ATR Signaling

**Figure 8:**
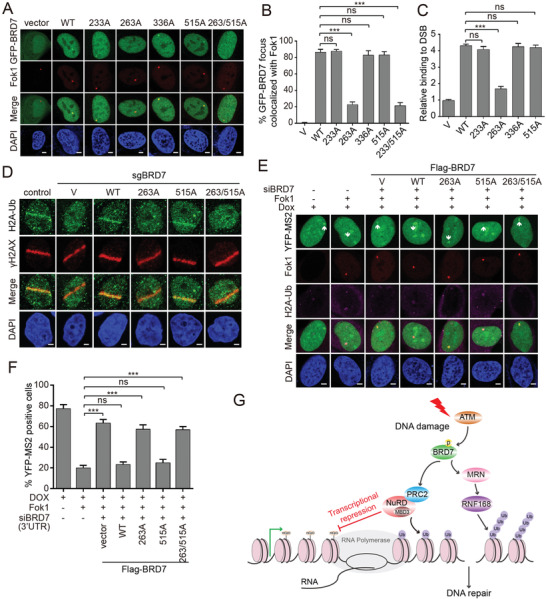
ATM‐mediated phosphorylation BRD7 at Ser 263 is required for HR repair and transcriptional repression. A) Recruitment of BRD7‐S263A to Fok1‐induced DSBs was remarkedly reduced. GFP‐BRD7 and indicated mutant constructs were transfected into U2OS‐265 DSB reporter cells for 24 h, and cells were induced site‐specific DSBs. Representative images after DNA damage are shown. B) Quantification of GFP‐BRD7 wild‐type and mutants focus colocalized with Fok1 of panel (A). Data are represented as mean ± SEM. Error bars indicate SEM. C) Flag‐BRD7 and mutants BRD7 recruitment to I‐SecI‐induced DSBs analyzed by ChIP assay. HeLa DR‐GFP reporter cells stably expressing indicated constructs were electroporated with I‐SecI plasmid. Eight hours after I‐SecI transfection, ChIP assay was performed to detect the enrichment of Flag‐BRD7 and mutants BRD7 relative to the vector control. D) Recruitment of H2A‐Ub to laser damage was reduced in BRD7‐S263A. Wild‐type and indicated mutant BRD7 were transfected into HeLa cells depleted of endogenous BRD7 and subjected to UV laser, and endogenous H2A‐Ub accumulation was analyzed by immunofluorescence. Representative images after DNA damage are shown. E) Transcription activity was measured in U2OS‐263 DSB reporter cells transfected with indicated BRD7 constructs after depletion of endogenous BRD7, followed by inducing site‐specific DSBs. Ongoing transcription of the reporter gene can be detected by the presence of a YFP‐MS2 foci. Representative images after DSBs induction are shown. F) Quantification of YFP‐MS2 positive cells from experiments as in panel (E). Quantification results are the average of three independent experiments and are shown as mean ± SEM. G) Model of the role of the PBAF subunit BRD7 in coordinating DSB‐induced transcriptional repression and HR repair. Scale bars, 2 µm. n.s., not significant; **P < 0.01, ***P < 0.001, Student's *t*‐test.

To investigate whether the effect of BRD7 on DSB‐induced transcriptional repression and HR repair are dependent on BRD7 phosphorylation, we initially examined the effect of BRD7 Ser 263 and Thr 515 phosphorylation on its accumulation to DSBs sites. As shown in **Figure** **8**A,B, recruitment of the phosphorylation‐resistant BRD7‐S263A mutant and BRD7‐S263A/T515A mutant to Fok1‐induced DSBs were markedly reduced, however, BRD7‐S233A, BRD7‐S336A and BRD7‐T515A mutants had no effect on recruitment of itself to DSBs. A ChIP assay with antibodies against Flag showed that BRD7‐S263A at the I‐SceI break site significantly decreased after I‐SceI‐induced DSBs, whereas mutants of BRD7‐S233A, BRD7‐S336A, and BRD7‐T515A were recruited to I‐SceI‐induced DSBs with an amounts similar to that of the wild‐type BRD7 (Figure 8C), suggesting that ATM‐mediated phosphorylation of BRD7 at Ser 263 is required for its recruitment to DNA damage sites. Consistent with the results of Figure S6H (Supporting Information), depletion of BRD7 dramatically decreased HR efficiency, and reconstitution of wild‐type BRD7, BRD7‐S233A BRD7‐S336A, but not BRD7‐S263A, BRD7‐T515A rescued these phenotypes (Figure S10A, Supporting Information). Thus, these findings demonstrate that ATM phosphorylates BRD7 on Ser 263 not only to facilitate its recruitment to DSBs but also to promote HR repair, and ATR‐mediated phosphorylation of BRD7 at Thr 515 is only required for proper HR but not for accumulation to DSBs sites.

Next, we reconstituted BRD7‐deficient cells with wild‐type BRD7 or indicated BRD7 mutants to further confirm the roles of BRD7 in DSB‐induced transcriptional repression (Figure S10B, Supporting Information). Wild‐type and mutant T515A of BRD7 rescued H2AK119Ub accumulation and exclusion of phosphorylation of RNAP II on Ser 2 at the sites of laser‐induced DSBs but not those expressing the BRD7‐S263A and BRD7‐S263A/T515A (Figure 8D and Figure S10C, Supporting Information). Moreover, we introduced the wild‐type and indicated mutants of BRD7 into U2OS‐263 cells depleted of endogenous BRD7, and found that the wild‐type and T515A mutant were able to rescue transcriptional repression after DSBs induction (Figure 8E,F). In contrast, the BRD7‐S263A and BRD7‐S263A/T515A were unable to restore Fok1‐induced transcriptional repression (Figure 8E,F). Mechanistically, both the BRD7‐S263A and BRD7‐S263A/T515A reduced its interaction with PRC2, NuRD, and MRN complexes as well as RNF168, while the BRD7‐T515A exhibited comparable affinity for these complexes compared with BRD7 wild‐type (Figure S10D, Supporting Information). These results suggested that the phosphorylation of BRD7 at Ser 263 by ATM is required for the recruitment of PRC2, NuRD and MRN complexes and RNF168 to DSBs sites and induction of transcriptional repression.

Further, we determined whether ATM and ATR signaling are affected by Ser 263 and Thr 515 phosphorylation and found that reconstitution of BRD7‐depleted cells with BRD7‐WT and BRD7‐T515A mutant, but not the BRD7‐S263A and BRD7‐S263A/T515A mutants, restored ATM Ser 1981 phosphorylation and activation of its targets CHK2 (Thr 68). Strikingly, neither S263A, T515A nor S263A/T515A mutants of BRD7 could rescue ATR Thr 1989 phosphorylation and phosphorylation of its substrate CHK1 (Ser 317) (Figure S10E, Supporting Information). These finding indicated that BRD7 Ser 263 phosphorylation is required for activation of both ATM/ATR signaling, whereas BRD7 Thr 515 phosphorylation by ATR kinase only contributes to ATR signaling.

## 3 Discussion

Here, we found that under the control of ATM, BRD7 (a specific subunit of PBAF complex) mediates DSB‐induced transcriptional repression and proper HR repair. Phosphorylation of BRD7 at Ser 263 by ATM kinase is required for direct interaction of BRD7 with PRC2/NuRD complex and subsequent accumulation of H2AK119Ub to DSB sites (Figures 1，2, 3, 4, and 8), consistent with previous finding that BRD7 can interact with PRMT5 and PRC2 to inhibit transcription of their target genes.^[^ ^56^ ^]^ This suggest that BRD7 may serve as a bridging factor to coordinate the ATM signaling and transcriptional repression regulated by PRC2/NuRD complexes.

Previous studies reported that the bromodomain (BRD) of BRD7 was responsible for the binding to histone H4 acetyl‐lysine, a mark associated with active transcription and DSBs.^[^ ^57^, ^58^ ^]^ We found that BRD7 is preferentially recruited to DSBs nearby transcriptionally active regions (Figure 2), and the endogenous BRD7 interacts with MBD3, which can recognize methylated DNA and trigger the formation of repressive chromatin, but not histone deacetylases HDAC1/2 (Figure 3A), suggesting that BRD7 represses transcription, at least in part, through specific interactions with methylated DNA by binding to MBD3,^[^ ^36^, ^59^ ^]^ but not via association with HDAC1/2 to deacetylate histone H3/4 acetylation.^[^ ^35^ ^]^ Indeed, we found that depletion of either MBD2 or MBD3 greatly rescued the expression of YFP‐MS2 at Fok1‐induced DSB sites, indicating that DNA methylation also contributes to DSB‐induced transcription silencing (Figure 3I,J) and DNA methyltransferase DNMT1 may fulfill this roles.^[^ ^60^ ^]^ Collectively, BRD7 may promote DSB‐induced transcriptional repression via PRC2‐mediated monoubiquitylation of H2AK119 as well as recruitment of the NuRD complex to CpG‐methylated DNA.

Previous study reported that histone acetyltransferases TIP60 activity was required for activation of ATM kinase and DNA damage‐induced transcriptional repression.^[^ ^61^, ^62^ ^]^ Based on these data, we propose a model that TIP60 is activated after DSB induction and leads to histone acetylation at DSB sites that is recognized by BRD7 to recruit the NuRD (containing MBD3, but not contained HDAC1/2) and PRC2 complexes (Figure 3A–H). These complexes further read and modify chromatin and chromatin‐associated proteins to form a compacted chromatin structures adjacent to the breaks, and promote transcriptional repression to subsequently facilitate DNA repair.

In the present study, we demonstrate the requirement of BRD7 for HR repair and early recruitment of the MRN complex (DNA ends sensor) and later stage recruitment of RNF168 to DSB flanking chromatin (Figures 4 and 5 and Figure S5, Supporting Information). We found that this process was governed by ATM kinase‐mediated phosphorylation of BRD7 at Ser 263 site and enhance the amplification of MRN/ATM signaling cascade to facilitates the HR repair (Figure 8A–C and Figure S10, Supporting Information). This phosphorylation event may induce a direct interaction of BRD7 with MRN complex and E3 ubiquitin ligase RNF168 leading to H2AK13/15Ub and NBS1 accumulation to DSBs, which in turn promote activation of ATM and more BRD7 phosphorylation (Figure 4E,F and Figure S10D,E, Supporting Information). This positive feedback loop at the early stage of DDR results in the amplification of MRN/ATM signaling cascade. The formation of *γ*H2AX by ATM recruits the large scaffold protein MDC1 to DSBs, promoting the recruitment of MRN complex and RNF8, RNF168 to the flanking chromatin of DSB at later stage of DDR.^[^ ^63^, ^64^ ^]^ Our results also showed that BRD7 could directly interact with RNF168 but not with RNF8 and is required for RNF168 recruitment to DSBs (Figure 4), suggesting that MRN complex‐independent recruitment of RNF168 may contribute to later stage of DDR.

It has been reported that in response to DSBs, MRN/ATM‐dependent DNA end resection leads to formation of ssDNA fractions resulting in ATR activation and subsequent Chk1 phosphorylation by ATR, which is called ATM‐to‐ATR switch.^[^ ^65^, ^66^ ^]^ Our study demonstrated that BRD7 depletion generates a significant decrease in Chk1 phosphorylation in response to either CPT or IR (Figure 6I,J and Figure S8G,H, Supporting Information). Meanwhile, we observed a moderate decrease in Chk1 phosphorylation at Ser 317 in BRD7 S263A cells upon either CPT or IR treatment (Figure 6I,J and Figure S10E, Supporting Information). We reasoned that the residual Chk1 phosphorylation in the absence of wildtype BRD7 may result from an incomplete inhibition of DNA end resection as indicated by a residual expression of phospho‐RPA2 (Figure S10E, Supporting Information). Several studies showed that the generation of RPA‐coated ssDNAs is an intermediate step not only for HR repair but also for ATR/Chk1 activation.^[^ ^67^, ^68^ ^]^ We found that BRD7 depletion also inhibited HU‐induced RPA2 focus formation, suggesting that BRD7 depletion impairs RPA2 recruitment in response to DNA damage (CPT or IR), as well as replication stress (Figure 6C,D). Indeed, we also found that phosphorylation of BRD7 at Thr 515 by ATR was important for the activation of ATR‐CHK1 signaling pathway but not for ATM‐CHK2 pathway (Figure S10A,E, Supporting Information). Loss of BRD7 phosphorylation at Thr 515 significantly reduced phosphorylation of ATR at Thr 1989 and expression of phospho‐RPA2, resulting in suppression of HR repair (Figure S10A,E, Supporting Information), consistent with previous studies implicated that the ATR‐CHK1 signaling pathway promotes HR repair.^[^ ^69^ ^]^ These results demonstrated that BRD7 depletion impairs DNA‐end resection not only ATM‐dependent but also ATR‐dependent in response to different types of genotoxic stress.

In conclusion, we propose the following mechanism about switching off transcription during DSB repair: Phosphorylation of BRD7 at Ser 263 by activated ATM facilitates interaction of BRD7 with PRC2 and NuRD complex and recruitment of these factors to DSBs sites flanking transcription active regions. Consequently, BRD7/ PRC2/NuRD complex induces transcriptional repression and enables the access of DSB repair factors to DSB sites. Meanwhile, phosphorylation of BRD7 at Ser 263 is also required for MRN complex and ubiquitin E3 ligase RNF168 recruitment to DSB lesions, proper HR repair and activation of MRN/ATM signaling pathway, resulting in recruiting BRD7 itself to DSBs flanking chromatin at a later stage of DDR (Figure 8G). Therefore, our findings shed new insight into how ATM signaling orchestrate DSB‐induced transcriptional repression and DNA repair processes to maintain genome integrity.

## 4 Experimental Section

##### Cells Culture, Drug Treatments, and Transfection

HeLa, U2OS, HEK293T, and MDA‐MB‐231 cells were obtained from ATCC and cultured in DMEM (Life Technologies) supplemented with 10% fetal bovine serum (FBS, Life Technologies) with 5% CO_2_ at 37 °C. The U2OS‐DSB reporter cell line (U2OS‐263 and U2OS‐265) developed by the Greenberg group was used to monitor DSB‐induced transcriptional repression.^[^ ^26^ ^]^ Drug treatment: both U2OS reporter cells pretreated with inhibitors to ATM (5 × 10^−6^ m, KU55933, S1092, Selleck), ATR (1 × 10^−6^ m, VE‐821, S8007, Selleck), DNA‐PK (5 × 10^−6^ m, NU7441, S2638, Selleck), and PARP (1 × 10^−6^ m, BMN673, S7048, Selleck) for 1 h. Inducing site‐specific DSBs: U2OS‐265 cells were treated with 0.5 × 10^−6^ m Sheild1 (Clontech, 632 189) and 1 × 10^−6^ m 4‐hydroxytamoxifen (4‐OHT) (Selleck, S7827) for 4 h to induce mCherry‐Fok1 expression and site‐specific DSBs. Inducing nascent transcription: U2OS‐265 cells were incubated with 1 µg mL^−1^ doxycycline (DOX) (Selleck, S4163) for an additional 3 h to induce nascent transcription of the reporter gene. The transcription levels of the reporter gene from both untreated and treated cells can be measured by quantitative PCR using primers described in Tang et al. as followed:^[^ ^26^ ^]^ p2‐forward: 5′‐GCTGGTGTGGCCAATGC‐3′, p2‐reverse: 5′‐TGGCAGAGGGAAAAAGATCTCA‐3′. Plasmid transfections were performed using Lipofectamine 3000 (Life Technologies). siRNA transfection was performed using Lipofectamine RNAiMAX reagent (Life Technologies) according to its protocol and the sequences of indicated siRNA were shown in Table S1 (Supporting Information).

##### Plasmids Construction

Full‐length cDNAs coding for BRD7, RNF8, and RNF168 were obtained from cDNA of human HEK293 cell by PCR and were then cloned into pcDNA3.1 vector with indicated tag sequence. SFB‐BRD7 and GFP‐BRD7 were first subcloned into pDONR221 vector and then transferred into destination vectors with the indicated SFB tag or GFP tag using Gateway Technology (Invitrogen, Camarillo, CA, USA). Flag‐ATM was a gift from Stephen Elledge (addgene plasmid #43 907) and Flag‐ATR was a gift from Aziz Sancar (addgene plasmid #31 611). Mutations were introduced using the TaKaRa MutanBEST Kit (TaKaRa, D401, Japan) with following primers; BRD7‐S233A‐Forward 5′‐GCCCAGGAAAGAATTCAGAGCCT‐3′, BRD7‐S233A‐Reverse 5′‐AAGAATTTTCATTCCTGAGTG‐3′, BRD7‐S263A‐Forward 5′‐GCACAGAGTGGGGAGGACGGAG‐3′, BRD7‐S263A‐Reverse 5′‐GGTGTCTGTTCCATCTTTCTG‐3′, BRD7‐S336A‐Forward 5′‐GCTCAGTGCGAATTTGAAAGA‐3′, BRD7‐S336A‐Reverse 5′‐GTTCACAAGCCGCCTGGTCAG‐3′, BRD7‐T515A‐Forward 5′‐GCTCAAGACAGGCTCATAGCGC‐3′, BRD7‐T515A‐Reverse 5′‐ACTGGAGTCCAAACGCCCTGG‐3′. All mutation sites were verified by DNA sequencing.

The NBS1 short hairpin RNA (shRNA) pLKO1 lentiviral constructs were purchased from Sigma Aldrich. The NBS1 targeting sequences are: 5′‐ GGAAGAAACGTGAACTCAA‐3′. The control sequence is: 5′‐ CCCATAAGAGTAATAATAT‐3′. The shRNAs were packaged into lentiviruses together with packaging plasmids pMG2G and pSPAX2 by transfecting into 293T cells. The HeLa cells were infected with lentiviruses supernatant followed by selection with media containing puromycin (2 µg mL^−1^) for 72 h.

##### Preparation of Chromatin Fractions

The collected cells (2 × 10^6^) were washed once with PBS and resuspended in 200 µL hypotonic buffer (10 × 10^−3^ m HEPES (pH 7.9), 10 × 10^−3^ m KCl, 1.5 × 10^−3^ m MgCl_2_, 0.34 m sucrose, 10% glycerol, 1 × 10^−3^ m DTT, 10 × 10^−3^ m NaF, 0.1% Triton X‐100 and protease inhibitors) for 5 min on ice, followed by low‐speed centrifugation (1300 g at 4 °C, 5 min) to separate the cytoplasmic proteins. The soluble nuclei were then resuspended in 200 µL extraction buffer (3 × 10^−3^ m EDTA, 0.2 × 10^−3^ m EGTA, 1 × 10^−3^ m DTT and protease inhibitors) and collected by centrifugation (1700 g at 4 °C, 5 min). After washing once with 200 µL extraction buffers, the insoluble chromatin was resuspended in 60 µL HCl (0.2 m) for 15 min on ice. The soluble chromatin‐binding proteins were neutralized with 60 µL Tris‐HCl (1 m, pH 8.0) and collected by high speed centrifugation (10 000 g at 4 °C, 5 min).

##### Laser Microirradiation and 5‐EU Assay

DNA double‐strand breaks were introduced in cultured cells with a 365 nm UV micro‐irradiation using a Zeiss Axiovert equipped with LSM 520 Meta. Briefly, cells cultured on glass‐bottomed dishes were pre‐sensitized with 10 × 10^−6^ m BrdU for 24 h. A 365 nm laser was carried out to generate BrdU‐dependent DSBs along the laser track. Ten minutes later, cells were pre‐extracted with PBS buffer containing 0.5% Triton X‐100 for 5 min followed by 3% paraformaldehyde for 15 min at room temperature. Cells were further incubated with indicated antibodies overnight after blocking with 5% BSA for 30 min at room temperature and subsequently subjected to immunofluorescence analysis as indicated in the Supporting Information.

For 5‐EU staining, U2OS cells were subjected to 365 nm UV laser followed by incubation with 1 × 10^−3^ m 5‐ethynyl uridine (5‐EU) at 37 °C for 1 h. In corporation of 5‐EU wad detected by Click‐iT RNA imaging kit (C10330, ThermoFisher Scientific) according to manufacturer's instructions. Samples were further immunostained for *γ*H2AX antibodies. Relative fluorescence values of 5‐EU were calculated by Image J software along the laser track highlighted by the *γ*H2AX staining. At least 20 cells were analyzed in each independent repeat.

##### Cells Synchronization

HeLa cells were synchronized at the G1/S boundary by performing a double thymidine block‐and‐release treatment as previously described,^[^ ^54^, ^70^ ^]^ and then were released into normal media at indicated time: 3 h (S‐phase cells), 6 h (G2‐phase cells), 9 h with incubation of 100 ng mL^−1^ nocodazole (M‐phase cells) and 11 h (G1‐phase cells). Cells were harvested and subjected to indicated experiments.

##### Co‐Immunoprecipitation and Western Blotting

Immunoprecipitation and western blotting were performed as described previously.^[^ ^55^ ^]^

Briefly, cells were lysed in RIPA buffer added with protease and phosphatase inhibitor (Bimake, China) and the clarified lysates were first incubated with either indicated antibodies or anti‐Flag‐agarose or anti‐S beads overnight at 4 °C, followed by precipitation being washed three times with NETN buffer (20 × 10^−3^ m Tris‐HCl, 100 × 10^−3^ m NaCl, 1 × 10^−3^ m EDTA, 0.5% Nonidet P‐40 and protease and phosphatase inhibitor). Then the samples were boiled in 2 × SDS loading buffer and resolved on SDS‐PAGE assay and transferred to PVDF membranes followed by blocking with 5% milk in PBST and incubation with indicated antibodies. The antibodies information were listed in Table S2 (Supporting Information).

##### Immunofluorescence Staining

Immunofluorescence was carried out as described previously.^[^ ^53^ ^]^ Cells grew on glass coverslips were treated with indicated drugs and then washed once with PBS followed by pre‐extraction with buffer containing 0.5% Triton X‐100 for 3 min and fixing with 3% paraformaldehyde for 10 min at room temperature. Cells were then incubated with indicated antibodies at 4 °C overnight and washed with PBS for three times followed by staining with secondary antibody at room temperature for 1 h. Cells were then stained with DAPI to visualize nuclear DNA and subjected to fluorescence microscope analysis.

##### HR, NHEJ, and SSA Repair Assays

HeLa cells stably integrating DR‐GFP (Addgene plasmid #26 475), EJ5‐GFP (Addgene plasmid #44 026) and SA‐GFP (Addgene plasmid #41 594) reporter respectively, were described previously.^[^ ^29^, ^43^, ^71^ ^]^ Briefly, 1 × 10^6^ HeLa reporter cells were electroporated with 10 µg of pCBASceI (Addgene plasmid #26 477) plasmid at 270 V, 950 µF using a BioRad Genepulsar. Cell were harvested 48 h after electroporation and subjected to flow cytometry analysis to detect percentages of GFP positive cells, which reflects repair efficiency induced by DNA double strand breaks. Means were representative of three independent experiments.

##### ChIP Assays

HeLa DR‐GFP cells or U2OS‐265 reporter cells were treated with indicated treatments followed by fixing with 1% formaldehyde for 10 min at room temperature. Then glycine (0.125 m) was added for another 5 min to stop the cross‐linking. The indicated cells harvested were lysed in cell lysis buffer (5 × 10^−3^ m PIPES‐KOH (pH 8.0), 85 × 10^−3^ m KCl, 0.5% NP‐40) containing protease and phosphatase inhibitor for 10 min on ice. Nuclei were obtained by centrifugation at 2200 g for 5 min and subjected to resuspend in nuclear lysis buffer (50 × 10^−3^ m Tris (pH 8.0), 10 × 10^−3^ m EDTA, 0.5% SDS containing protease and phosphatase inhibitor) and sonicated to shear DNA to an average fragment size of 500 bp using Bioruptor XL sonicator (Diagenode). After centrifugation, the supernatant was diluted five times with dilution buffer (16.7 × 10^−3^ m Tris (pH 8.0), 1.2 × 10^−3^ m EDTA, 167 × 10^−3^ m NaCl, 0.01% SDS, 1% Triton X‐100 containing protease and phosphatase inhibitor). Ten percent of supernatant was used as input control and the remaining was divided equally to incubate with either IgG antibody or targeted proteins plus protein G agarose at 4 °C overnight. Then the beads were washed four times sequentially, once in low salt buffer (50 × 10^−3^ m Tris (pH 8.0), 1 × 10^−3^ m EDTA, 150 × 10^−3^ m NaCl, 0.1% SDS, 1% Triton X‐100, 0.5% deoxycholate), once in high salt buffer (50 × 10^−3^ m Tris (pH 8.0), 1 × 10^−3^ m EDTA, 500 × 10^−3^ m NaCl, 0.1% SDS, 1% Triton X‐100, 0.5% deoxycholate), once in LiCl buffer (50 × 10^−3^ m Tris (pH 8.0), 1 × 10^−3^ m EDTA, 250 × 10^−3^ m LiCl, 1% Triton X‐100, 0.5% deoxycholate) and once in ddH_2_O. The obtained beads were resuspended in elution buffer (0.1 M NaHCO_3_, 1% SDS) and rotate for 20 min at room temperature to elute protein‐DNA complex. The eluted samples were further incubated with 0.2 m NaCl at 65 °C for 8 h to reverse cross‐link followed by treatment with RNase A and proteinase K at 37 °C for 1 h. Finally, DNA was purified and subjected to quantitative PCR analysis. The value of enrichment was calculated according to the relative amount of input and the ratio to IgG. The ChIP‐PCR primers were as follows: For DR‐GFP cells, F1: 5′‐TTATTGTGCTGTCTCATCATT‐3′, R1: 5′‐GTGCTGCATGCTTCTTCGGCA‐3′, F2: 5′‐TCCATCTCCAGCCTCGGGGCT‐3′, R2: 5′‐AGGCTCTAGAGCCGCCGGTCA‐3′, control primers selected against a specific region of chromosome 12 were F: 5′‐ATGGTTGCCACTGGGGATCT‐3′, R: 5′‐TGCCAAAGCCTAGGGGAAGA‐3′. For U2OS‐265 reporter cells, F1: 5′‐GCTGGTGTGGCCAATGC‐3′, R1: 5′‐TGGCAGAGGGAAAAAGATCTCA ‐3′.^[^ ^26^ ^]^

##### Phospho‐Histone H3 and Phospho‐Histone H2AX Staining

The procedure was performed as described previously.^[^ ^53^, ^55^, ^70^ ^]^ Briefly, cells were incubated with CPT (10 × 10^−9^ m) for 1 h or left untreated and subsequently grown in the presence of paclitaxel (Taxol; 2 × 10^−6^ m) for indicated times and then the cells were harvested and fixed in 70% ethanol at −20 °C overnight. The cells were resuspended in 1 mL of 0.25% Triton X‐100 in PBS, and rotated at 4 °C for 15 min. After the cells were centrifuged, the cell pellet was suspended in 100 µL of PBS containing 1% bovine serum albumin (BSA) and 2 µg Phospho‐Histone H3 (Ser 10) or Phospho‐Histone H2AX (Ser 139) antibodies, and incubated for 2 h at room temperature. Then, the cells were rinsed with PBS containing 1% BSA for two times and stained with propidium iodide (PI), and cellular fluorescence was measured using a FC‐500 flow cytometer (Beckman Coulter).

##### GST Pulldown Assays

The procedure was performed as previously described with minor modification.^[^ ^54^ ^]^ Briefly, GST‐BRD7 wildtype and indicated mutants were purified from bacteria and further immobilized on glutathione‐agarose beads (GE Healthcare). The beads were incubated with HeLa cell lysates overnight at 30 °C. Beads were then washed with NETN buffer three times, and bound proteins were analyzed by western blotting.

##### In Vitro ATM/ATR Kinase Assays

The procedure was performed as previously described with minor modification.^[^ ^70^, ^72^ ^]^ Briefly, ATM and ATR proteins were immunoprecipitated from HEK293T cells transfected with Flag‐ATM (addgene plasmid #43 907) and Flag‐ATR (addgene plasmid #31 611) constructs using anti‐flag agarose. GST only, GST‐BRD7 wild‐type and GST‐BRD7 various mutants were purified from bacteria using glutathione agarose beads (GE Healthcare). The sample were then incubated with Flag‐ATM or Flag‐ATR proteins in 50 µL of kinase buffer (Cell Signaling Technology) containing 10 × 10^−6^ m ATP for 30 min at 30 °C. Reactions were terminated by addition of 2 × sample buffer and subsequently analyzed by Western blotting using indicated antibodies.

##### Cell Survival Assays

Control or sgRNAs treated cells (1 × 10^3^) were divided into six well dishes in triplicates. Cells were then treated with CPT or PARP inhibitor (Olaparib, Rucaparib, BMN673) at indicated concentrations. Cells were then incubated for 14 days, the resulting colonies were fixed and stained with Crystal Violet.

## Conflict of Interest

The authors declare no conflict of interest.

## Author Contributions

K.H., Y.L., W.W., and L.X. contributed equally to this work. E.S., D.Y., and K.H. designed the experiments. K.H. wrote the draft manuscript. E.S., D.Y., and H.P.K. provided insightful discussion and revision for this manuscript. K.H., Y.L., W.W., and L.X. carried out most experiments and analyzed data. Y.C., H.Y., Z.C., Y.Z., Q.J., L.L., and J.L. assisted in the experiments.

## Supporting information

Supporting Information

Supplementary Table 1
