# Supplementary material for: ATM‐Dependent Recruitment of BRD7 is required for Transcriptional Repression and DNA Repair at DNA Breaks Flanking Transcriptional Active Regions
Source: Adv Sci (Weinh). 2020 Sep 3;7(20):2000157. doi: 10.1002/advs.202000157 (PMC7578904; doi:10.1002/advs.202000157)
Supplement: Supplementary file 1 — Supporting Information [file ADVS-7-2000157-s001.pdf]

WILEY-VCH

© 2020 Wiley-VCH GmbH

ADVANCED SCIENCE
Open Access

Supporting Information

for *Adv. Sci.*, DOI: 10.1002/advs.202000157

ATM-dependent Recruitment of BRD7 is Required for Transcriptional Repression and DNA Repair at DNA Breaks Flanking Transcriptional Active Regions

Kaishun Hu, Yu Li, Wenjing Wu, Limin Xie, Haiyan Yan, Yuexin Cai, Dong Chen, Qiongchao Jiang, Lehang Lin, Zhen Chen, Jian-You Liao, Yin Zhang, H. Phillip Koeffler, Dong Yin and Erwei Song**

© 2020 Wiley-VCH GmbH

Supporting Information

ATM-dependent recruitment of BRD7 is required for transcriptional repression and DNA repair at DNA breaks flanking transcriptional active regions

Kaishun Hu[1#], Yu Li[1#], Wenjing Wu[1,2#], Limin Xie[1#], Haiyan Yan[1], Yuexin Cai[1], Dong Chen[1], Qiongchao Jiang[1,3], Lehang Lin[1], Zhen Chen[1], Jian-You Liao[1], Yin Zhang[1], H. Phillip Koeffler[4], Dong Yin [1] and Erwei Song[1,2]**

ATM-dependent recruitment of BRD7 is required for transcriptional repression and DNA repair at DNA breaks flanking transcriptional active regions

Kaishun Hu[1#], Yu Li[1#], Wenjing Wu[1,2#], Limin Xie[1#], Haiyan Yan[1], Yuexin Cai[1], Dong Chen[1], Qiongchao Jiang[1,3], Lehang Lin[1], Zhen Chen[1], Jianyou Liao[1], Yin Zhang[1], H. Phillip Koeffler[4], Dong Yin [1]* and Erwei Song[1,2]**

Figure S1

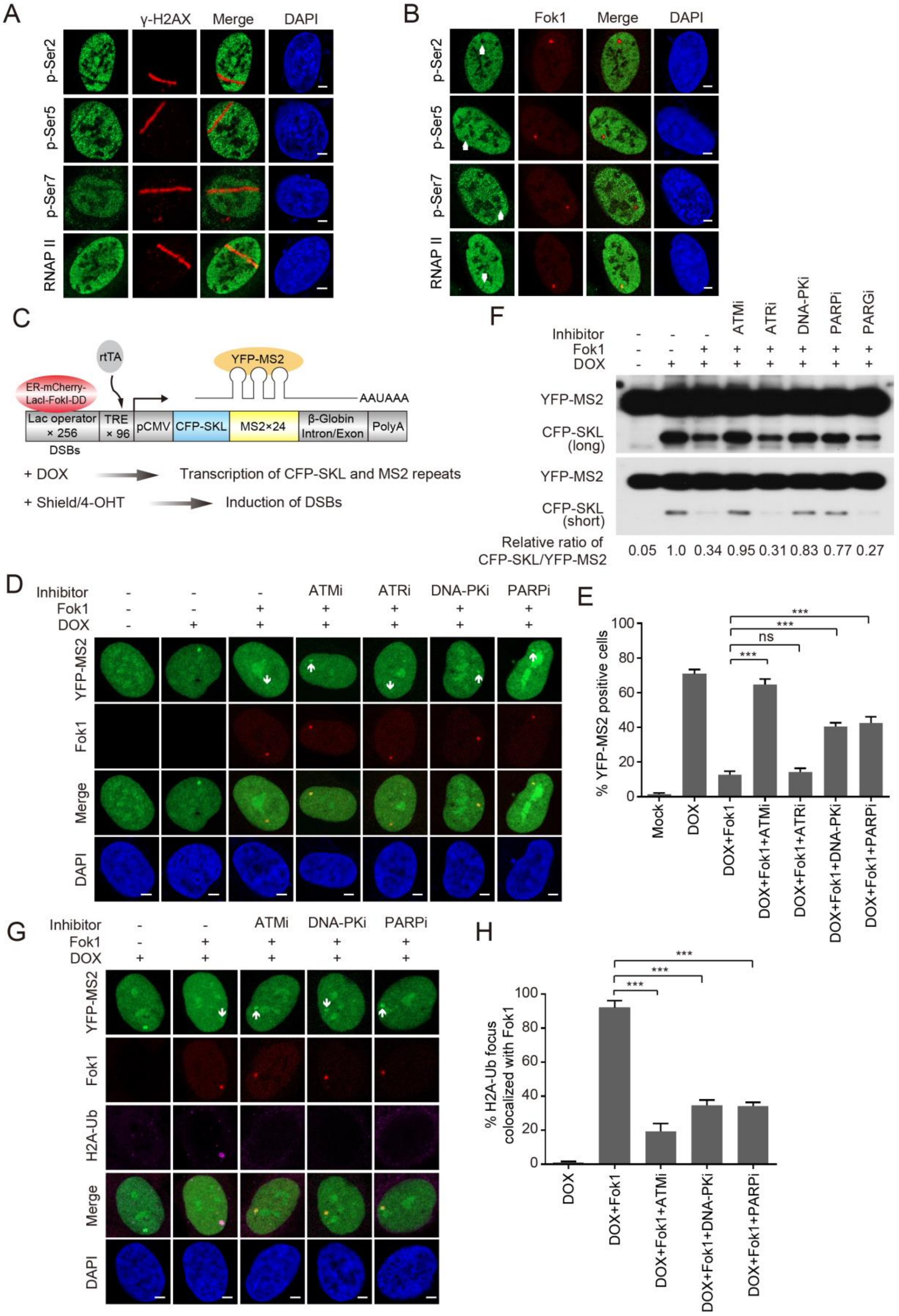

A
γ-H2AX
Merge
DAPI
p-Ser2
p-Ser5
p-Ser7
RNAP II
B
Fok1
Merge
DAPI
p-Ser2
p-Ser5
p-Ser7
RNAP II
C
rtTA
YFP-MS2
ER-mCherry-LacI-FokI-DD
AAUAAA
Lac operator × 256
TRE × 96
pCMV
CFP-SKL
MS2×24
β-Globin Intron/Exon
PolyA
DSBs
+ DOX
Transcription of CFP-SKL and MS2 repeats
+ Shield/4-OHT
Induction of DSBs
F
Inhibitor
Fok1
DOX
ATMi
ATRi
DNA-PKi
PARPi
PARGi
YFP-MS2
CFP-SKL (long)
YFP-MS2
CFP-SKL (short)
Relative ratio of CFP-SKL/YFP-MS2
0.05 1.0 0.34 0.95 0.31 0.83 0.77 0.27
D
Inhibitor
Fok1
DOX
ATMi
ATRi
DNA-PKi
PARPi
YFP-MS2
Fok1
Merge
DAPI
E
% YFP-MS2 positive cells
***
***
ns
***
Mock
DOX
DOX+Fok1
DOX+Fok1+ATMi
DOX+Fok1+ATRi
DOX+Fok1+DNA-PKi
DOX+Fok1+PARPi
G
Inhibitor
Fok1
DOX
ATMi
DNA-PKi
PARPi
YFP-MS2
Fok1
H2A-Ub
Merge
DAPI
H
% H2A-Ub focus colocalized with Fok1
***
***
***
DOX
DOX+Fok1
DOX+Fok1+ATMi
DOX+Fok1+DNA-PKi
DOX+Fok1+PARPi

Figure S1. ATM, DNA-PKs and PARP1 are required for transcriptional repression at DNA double strand breaks. (A) Actively elongating RNA polymerase II was inhibited at sites of laser damage in U2OS cells using antibodies against p-Ser 2, p-Ser 5 and p-Ser 7 of the C-terminal domain of the RNAP II large subunit. (B) Immunofluorescence was performed for each group as indicated in Figure S1B and immunostained with indicated antibodies. (C) Schematic of the U2OS-263 DSB reporter system that allows monitoring transcription following induction of DSBs. Shield-1 and 4-OHT regulate the expression of Fok1 nuclease, which induces DSBs upstream of CFP-SKL genes within Lac operator. Doxycycline induces transcription of the reporter gene CFP-SKL containing MS2 stem loop, allowing visualization by the accumulation of the YFP-MS2 protein. (D) U2OS-263 reporter cells treated with either DMSO, ATM inhibitor (Ku55933, 10 μM), ATR inhibitor (VE-821, 10 μM), DNA-PK inhibitor (NU7441, 5 μM) or PARP inhibitor (BMN673, 10 μM) for 2 h, followed by the addition of Shield-1 (0.5 μM) and 4-OHT (1 μM) for additional 4 h. Ongoing transcription of the reporter gene can be detected by the presence of a YFP-MS2 foci. Representative images after DSBs induction are shown in D. Scale bars, 2 μm. (E) Quantification of YFP-MS2 positive cells from experiments in panel D. (F) The cells indicated in D were lysed with RIPA buffer, and subjected to Western blot with the indicated antibodies. YFP-MS2 levels are used as a loading control. (G) DSB-induced H2AK119Ub (H2A-Ub) at transcription sites was reduced in U2OS-263 reporter cells treated with ATM, DNA-PK and PARP inhibitor. Representative images after DSBs induction are shown in G. Scale bars, 2 μm. (H) Quantification results of H2A-Ub foci positive cells from experiments in panel G. All the quantification results are the mean of three independent experiments and shown as mean ± SEM. n.s., not significant; $**P < 0.01$, $***P < 0.001$, Student's t-test.

Figure S2

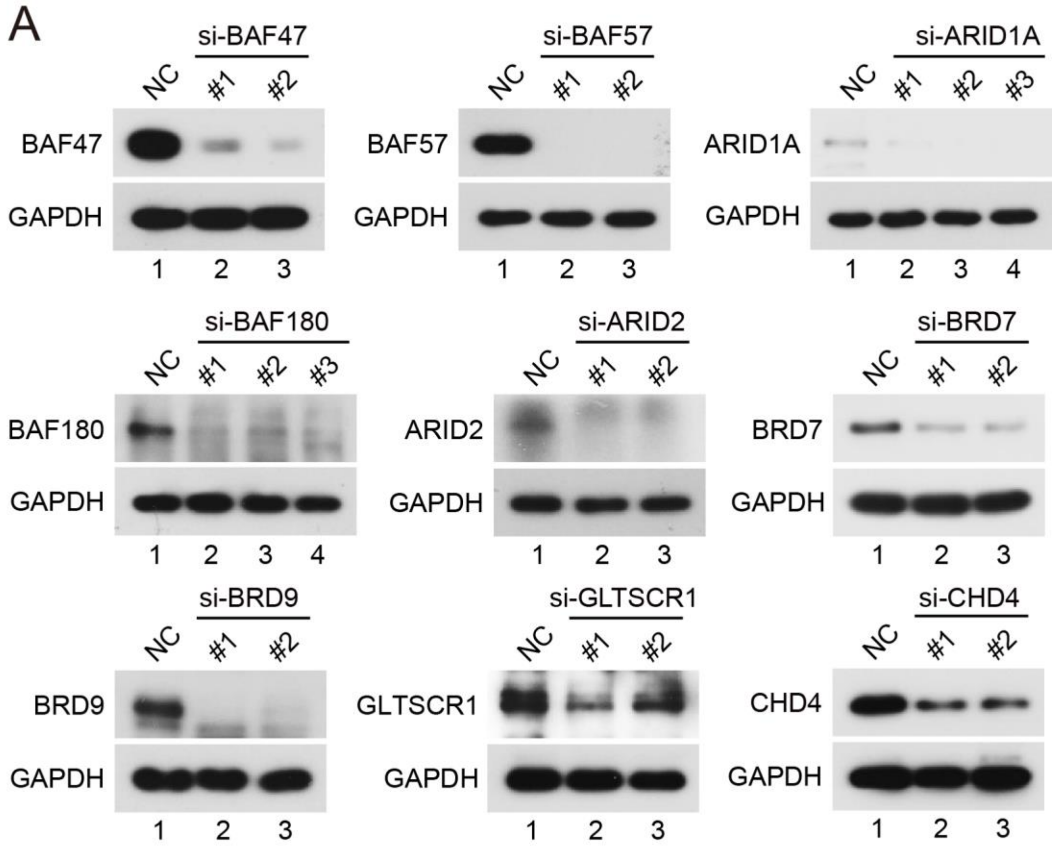

Figure S2. Validation of the indicated siRNA targeting BAF, PBAF, ncBAF and NuRD complexes. (A) Western blot analysis of whole-cell extracts prepared from U2OS cells treated with siRNA targeting BAF complex (BAF47, BAF57, ARID1A), or PBAF complex (BAF180, BRD7, ARID2), or ncBAF complex (GLTSCR1), or NuRD complex (CHD4). NC, negative control. GAPDH was used as a loading control.

Figure S3

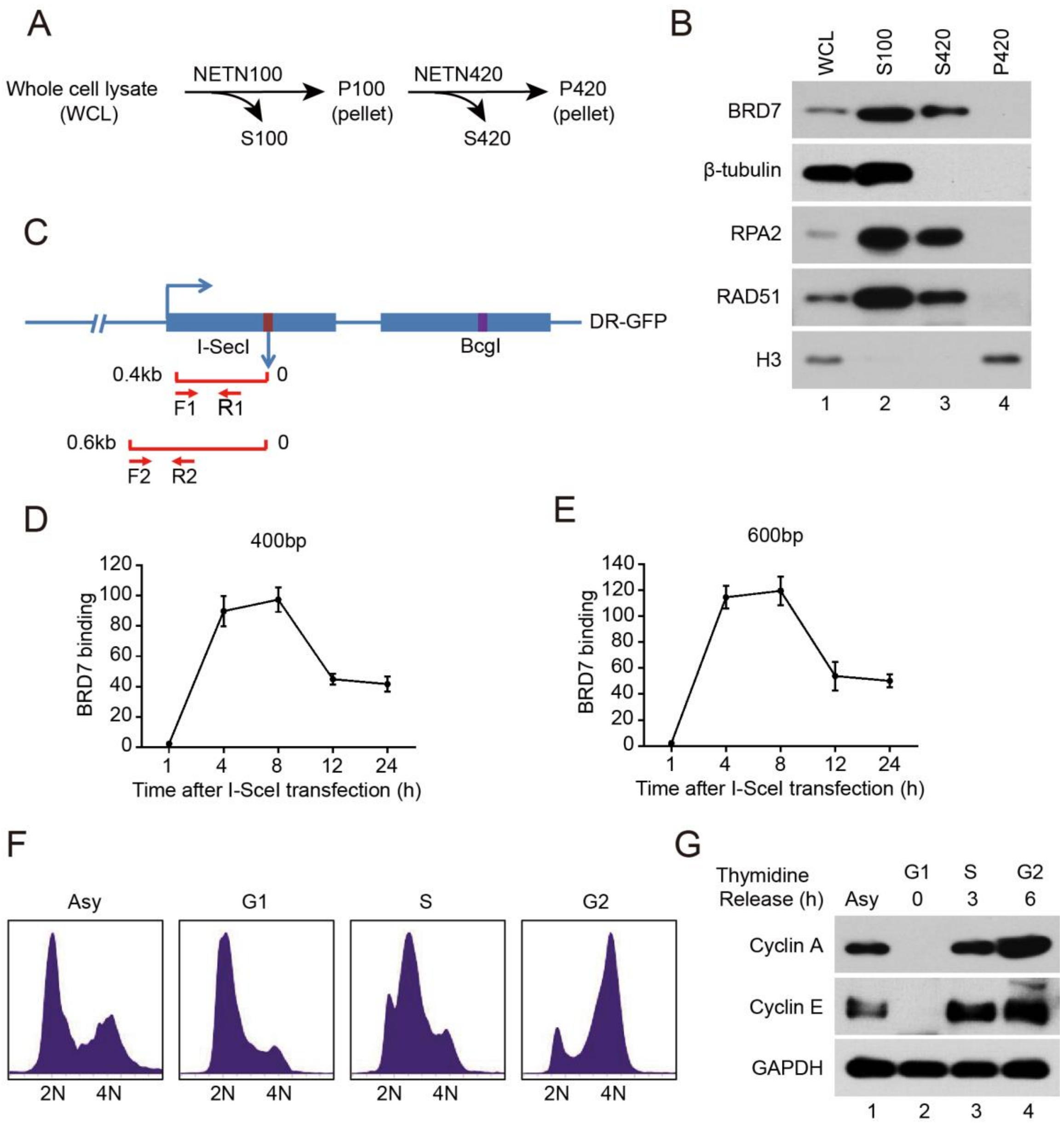

Figure S3. BRD7 is a chromatin-associated protein and involved in cellular response to DNA damage. (A) Schematic diagrams of the fractionation procedures of chromatin binding proteins. (B) BRD7 tightly associated with chromatin. HeLa cells were lysed sequentially in NETN buffer with increasing concentrations of salt. Cell lysates were then subjected to Western blot with indicated antibodies. (C) DR-GFP construct containing I-SceI restriction enzyme was stably integrated into HeLa cells, and the indicated primers covering a range of distances from the cutting site were used for the PCR. (D and E) HeLa cells carrying the DR-GFP reporter were transfected with I-SceI plasmids. ChIP analyses with BRD7 or control IgG antibodies were performed at the indicated time points after I-SceI transfection. The indicated

primers F1/R1 (D) or F2/R2 (E) were used for the ChIP-PCR. The data represent the mean ± SEM of three experiments and Error bars indicate SEM. (F and G) HeLa DR-GFP cells were treated with double-thymidine to achieve cells at G1-S boundary and then released at indicated time points: 0 h (G1 phase), 3 h (S phase), 7 h (G2 phase). The cells harvested at indicated phases were subjected to flow cytometer (F) or western blot with indicated antibodies (G) according to the Experimental Procedures.

Figure S4

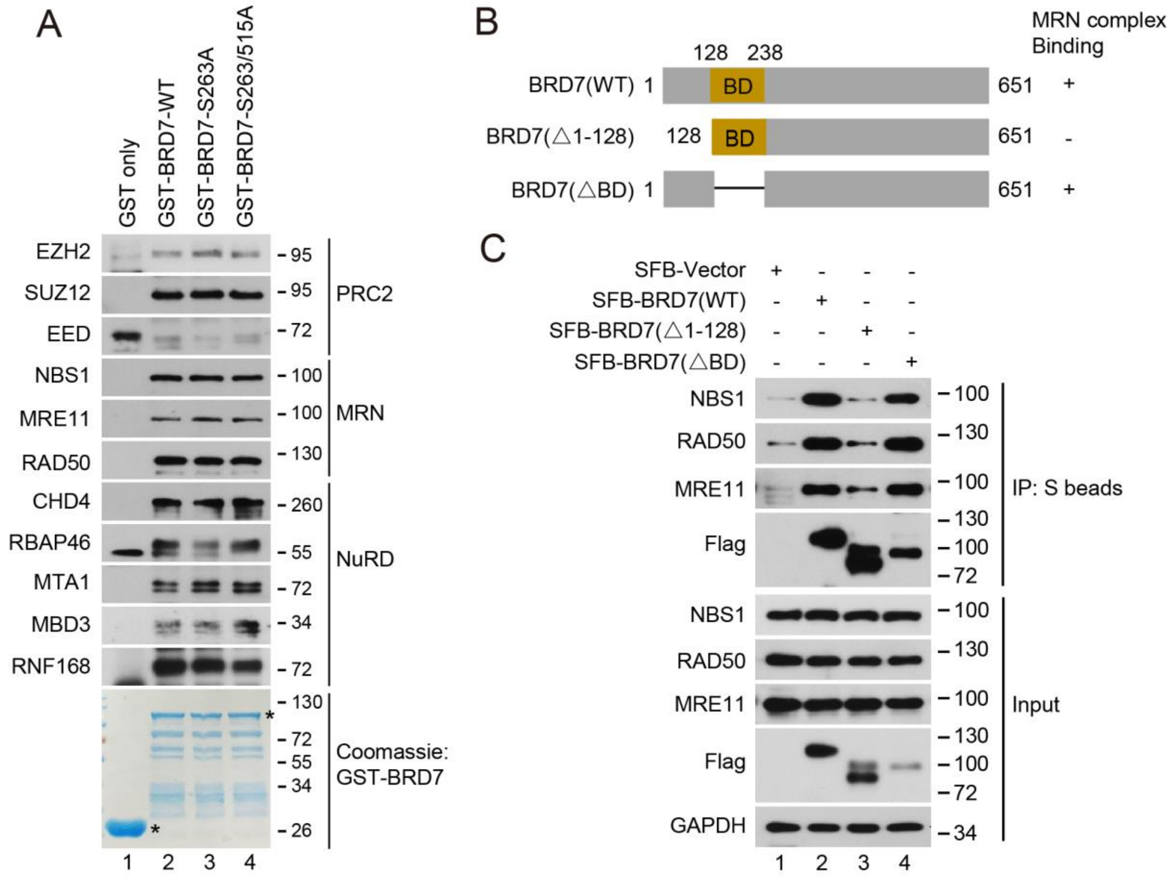

Figure S4. BRD7 directly interacts with PRC2, NuRD and MRN complex, and the amino-terminal part of BRD7 (residues 1-128) is required for the interaction between BRD7 and the MRN complex. (A) BRD7 directly interacts with PRC2, NuRD and MRN complex by performing GST pull-down assays. HeLa cell lysate was incubated with either GST-alone beads or the indicated GST-BRD7 beads at 4℃ overnight. Lower panel: Purified proteins visualized by Coomassive staining. An asterisk (*) indicates the corresponding BRD7 fragments or GST only fragment. (B) Schematic depiction of the domains of BRD7. (C) HeLa cells were transfected with SFB-BRD7 wild-type and indicated mutants of BRD7 for 24 h, and then treated with IR (5 Gy) for 1 h. Cells were lysed with RIPA in the presence

of benzonase, followed by incubating with S beads, and Western blot analysis was performed with indicated antibodies.

Figure S5

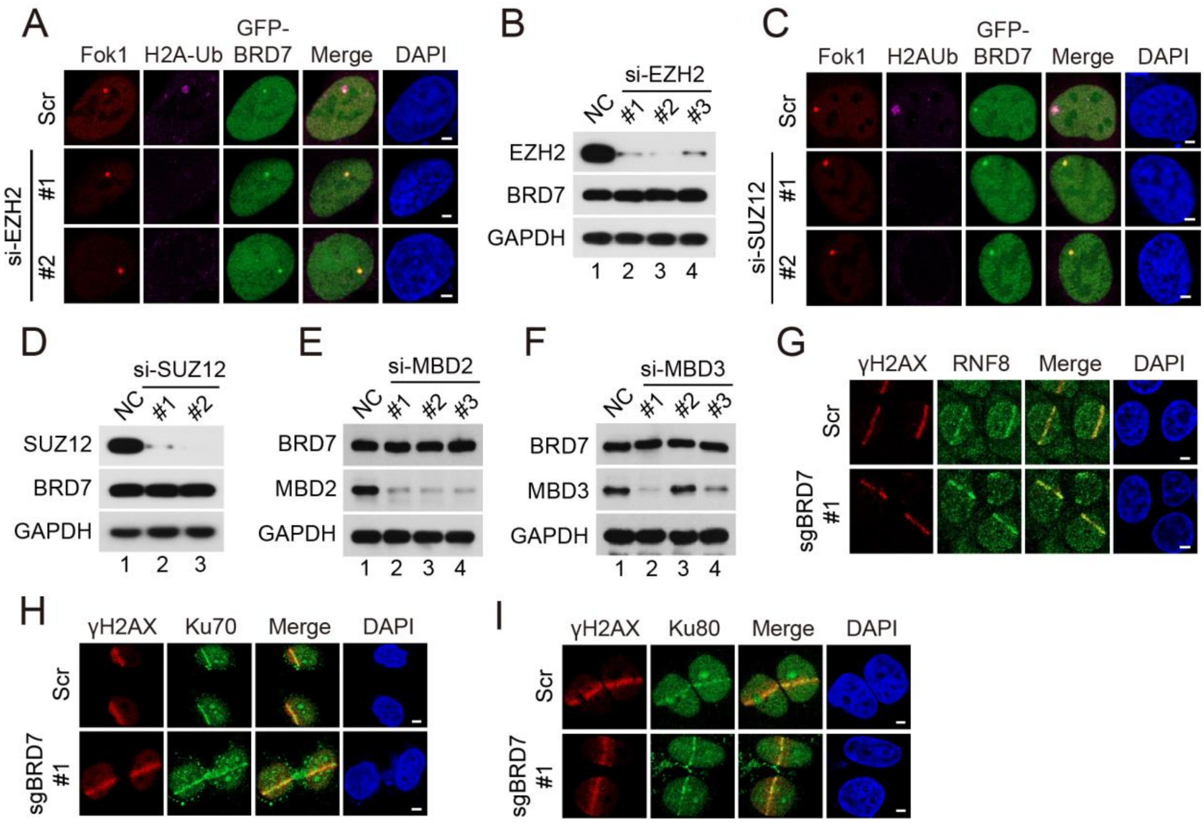

Figure S5. PRC2 complex is not required for the recruitment of BRD7 to Fok1-induced DSBs, and BRD7 is not required for the recruitment of ubiquitin E3 ligase RNF8 and NHEJ key factor Ku70 and Ku80. (A) GFP-BRD7 recruited to Fok1-induced DSBs in EZH2-depleted cells. The EZH2 siRNAs were transfected into U2OS-265 DSB reporter cells for 48 h following by transfection of GFP-BRD7 and cells were damaged by inducing site-specific DSBs, followed by analysis of immunofluorescence. Representive images after DNA damage are shown. Scale bars, 2 μm. (B) U2OS-265 cells were transfected with either scrambled or EZH2 siRNAs for 48 h, protein levels were detected by Western blot with the indicated antibodies. (C) SUZ12 depletion did not affect the accumulation of GFP-BRD7 to Fok1-induced DSBs. The SUZ12 siRNAs were transfected into U2OS-265 cells for 48 h following by transfection of GFP-BRD7 and cells were subjected to site-specific DSBs, followed by analysis of immunofluorescence. Representive images after DNA damage are shown. Scale bars, 2 μm. (D) U2OS-265 cells were transfected with either scrambled or SUZ12 siRNAs for 48 h, and lysed with RIPA buffer, and lysates were subjected to Western blot. (E and F) Western blot analysis of whole-cell extracts prepared from U2OS-263 cells

treated with siRNA targeting MBD2 and MBD3. NC, negative control. GAPDH was used as a loading control. (G-I) BRD7 is not required for the recruitment of RNF8, Ku70 and Ku80 to laser damage site. Control and BRD7-depleted cells were subjected to UV laser, and endogenous RNF8, Ku70 and Ku80 accumulation were analysed by immunofluorescence. Representive images after DNA damage are shown. Scale bars, 2 μm.

Figure S6

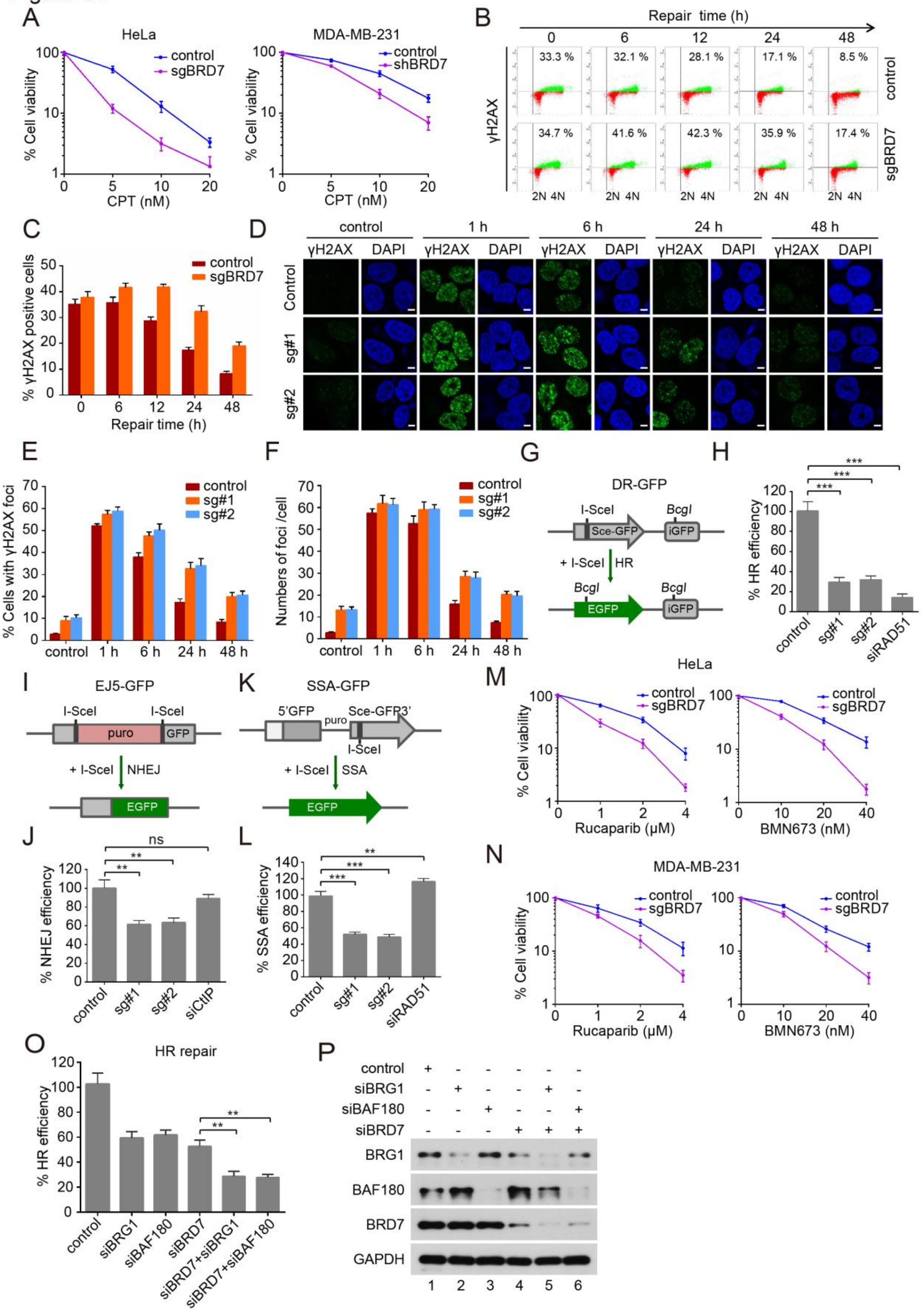

Figure S6. BRD7 is involved in cellular response to DNA damage and promotes HR

repair and NHEJ repair. (A) BRD7 depletion sensitizes cells to CPT. HeLa cells were infected with control or BRD7-specific lentiviral sgRNAs and cultured in medium containing puromycin. The stable knockout cell lines were treated with indicated doses of CPT for 1 h, and then supplied with fresh medium. After 14 days, cells were stained with Crystal Violet. Results shown are averages of three independent experiments. (B and C) BRD7 depletion inhibits DNA repair. Control or BRD7-depleted HeLa cells were either mock treated or treated with etoposide (5 μm) for 1 h and allowed to repair for the indicated time intervals. Flow cytometric analysis of DNA content and γH2AX level was obtained by using propidium iodide (PI) and Alexa Fluor 488 anti-γH2AX antibody. The data shown are from a single representative experiment out of three replicates. Quantification of γH2AX-positive cells is shown in C as the mean ± SEM. (D-F) BRD7 depletion inhibits DNA repair. Control or BRD7-depleted HeLa cells were either mock treated or treated with etoposide (5 μm) for 1 h and allowed to recover for 1, 6, 24, 48 h before fixing and processed for immunofluorescence using Alexa Fluor 488 anti-γH2AX antibody. Representative images of γH2AX foci in panel D. Scale bars, 5 μm. Quantification results of panel E and F are from the average of three independent experiments and are shown as mean ± SEM. More than 100 cells were counted in each group. (G). Schematic representation of HR reporter. (H) BRD7 depletion suppresses HR repair. HeLa DR-GFP cells were infected with the indicated sgRNAs or siRNA and 72 h later were electroporated with I-SceI plasmid. 48 h after transfection, cells were harvested and performed for GFP expression by flow cytometry analysis (FACS). (I) Schematic representation of NHEJ reporter. (J) BRD7 depletion impairs NHEJ efficiency. HeLa EJ5-GFP cells were infected with the indicated sgRNAs or siRNA and 72 h later were electroporated with I-SceI plasmid. 48 h after transfection, cells were harvested and performed for GFP expression by flow cytometry analysis (FACS). (K) Schematic representation of SSA reporter. (L) BRD7 depletion inhibits SSA repair. HeLa SA-GFP cells were infected with the indicated sgRNAs or siRNA and 72 h later were electroporated with I-SceI plasmid. 48 h after transfection, cells were harvested and performed for GFP expression by flow cytometry analysis (FACS). Quantification results are the average of three independent experiments and are shown as mean ± SEM. (M and N) HeLa or MDA-MB-231 cells were infected with indicated sgRNA, and selected with puromycin for 4 days. The resulting cell lines with BRD7 knockout were treated with indicated doses of Rucaparib or BMN673, and cell survival assays were performed. Results shown are averages of three independent experiments. (O and P) HeLa DR-GFP cells were infected with the indicated

siRNAs alone or in combination and 72 h later were electroporated with I-SceI plasmid. 48 h after transfection, cells were harvested and performed for GFP expression by flow cytometry analysis (FACS) and western blot analysis (P). n.s., not significant; **P < 0.01, Student's t-test.

Figure S7

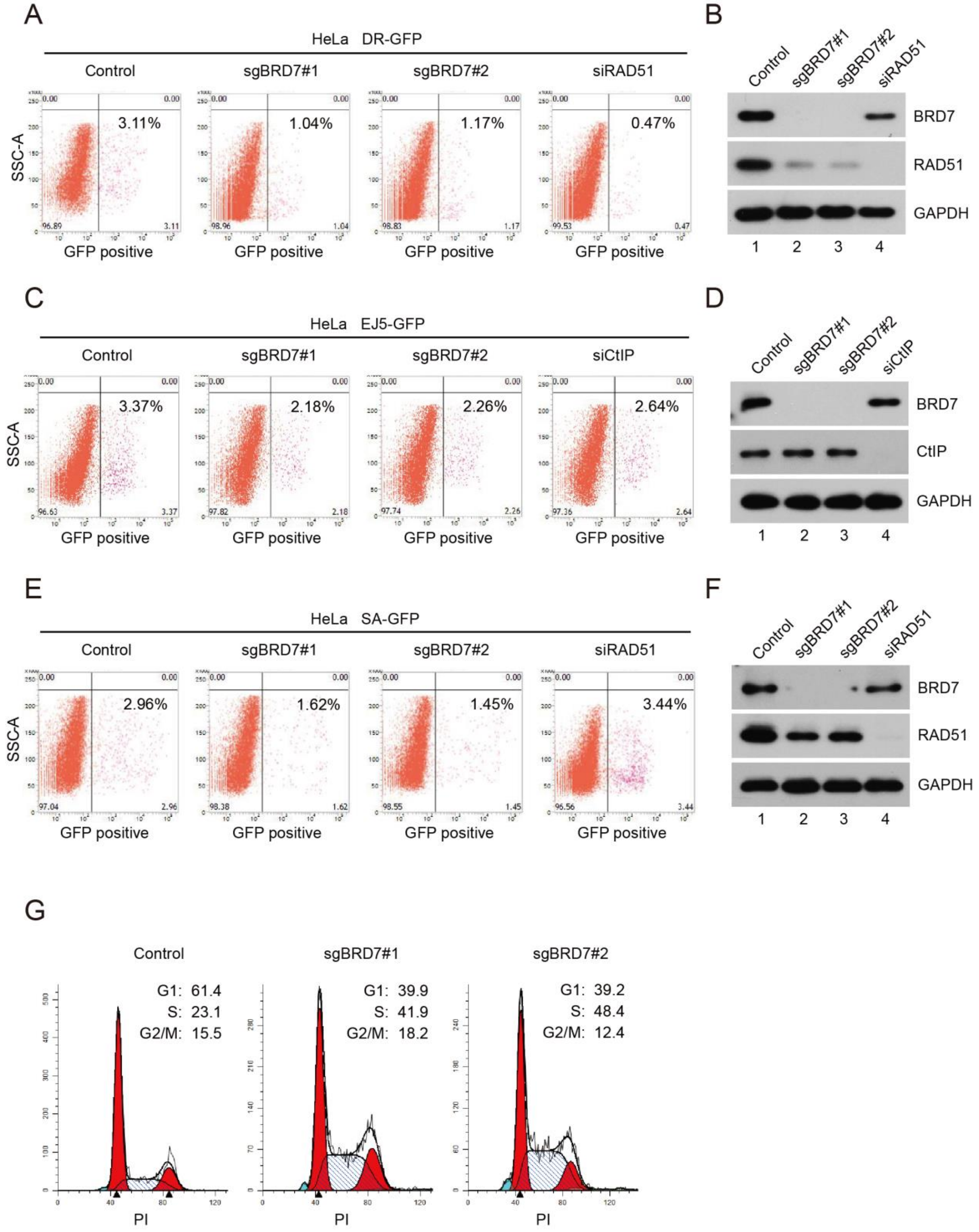

Figure S7. BRD7 promotes both the HR and NHEJ efficiencies

(A and B) BRD7 depletion impairs HR repair. HeLa DR-GFP cells transfected with the indicated sgRNAs and RAD51 siRNAs were electroporated with an I-Sce1 plasmid. 48 h after electroporation, cells were harvested and subjected to flow cytometry (FACS) and western blot analysis. Representative images of GFP expression were shown in A. (C and D) BRD7 depletion inhibits NHEJ repair. HeLa EJ5-GFP cells transfected with the indicated sgRNAs and CtIP siRNAs were electroporated with an I-Sce1 plasmid. 48 h after electroporation, cells were harvested and subjected to perform for GFP expression by FACS and western blot analysis. Representative images of GFP expression were shown in C. (E and F) BRD7 depletion inhibits SSA repair. HeLa SA-GFP cells transfected with the indicated sgRNAs and RAD51 siRNAs were electroporated with an I-Sce1 plasmid. 48 h after electroporation, cells were harvested and subjected to FACS and western blot analysis. Representative images of GFP expression were shown in E. (G) BRD7 depletion promotes G1-S transition. HeLa cells depleted of endogenous BRD7 were harvested and fixed with ice-clod 70% ethanol overnight. The cells were washed with PBS three times and incubated with RNase A (200 μg/ml) for 20 min, followed by propidium iodide (PI) (25 μg/ml) staining and FACS analysis.

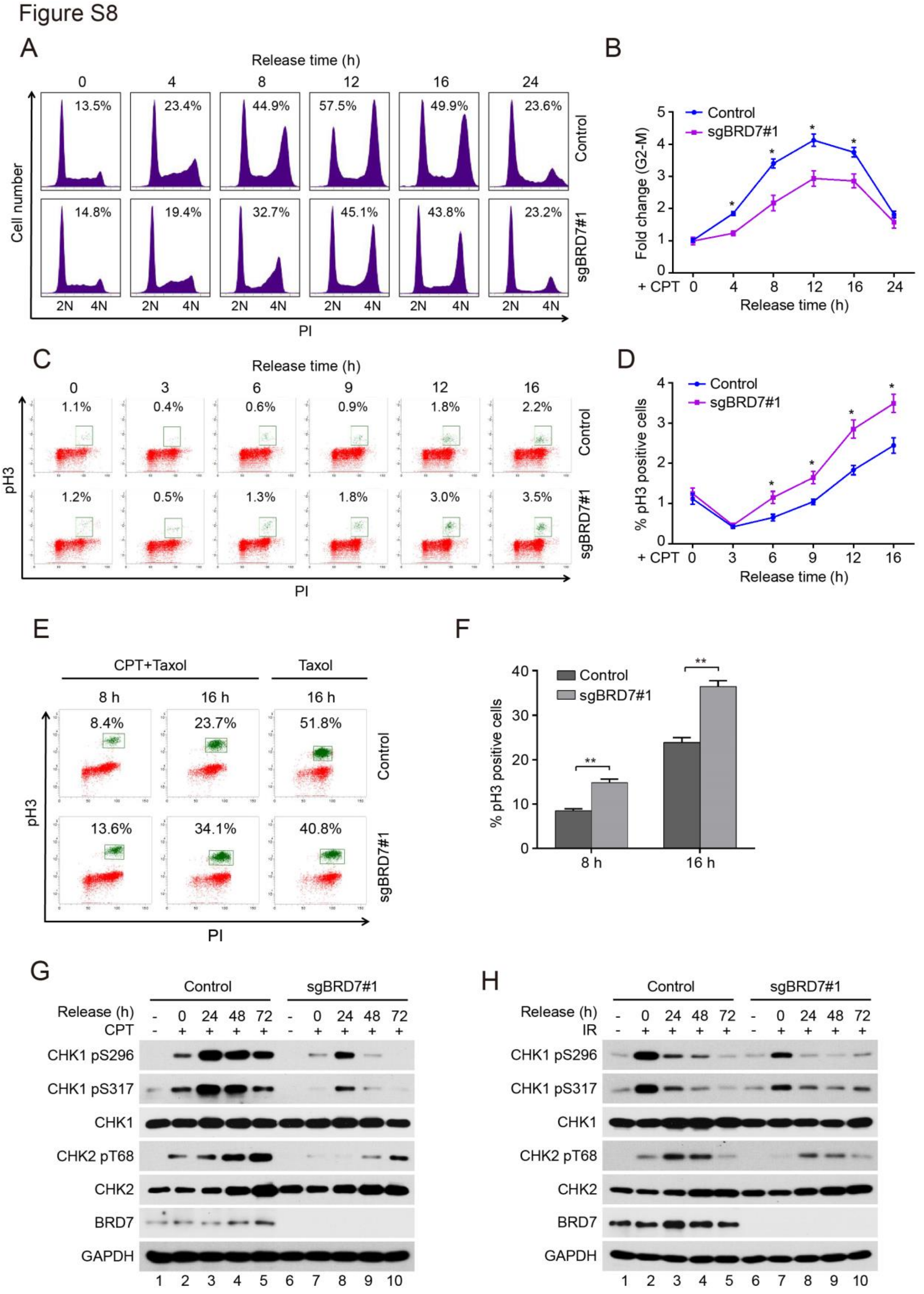

Figure S8. Depletion of BRD7 impairs G2-M DNA damage checkpoint.

(A and B) Control or BRD7-depleted HeLa cells were treated with CPT (10 nM) for 1 h and

subsequently released the cells into fresh medium lacking CPT to allow cells to repair for indicated time intervals. Flow cytometric analysis of DNA content was obtained by using propidium iodide (PI). The data shown are from a single representative result out of three replicates. (B) Relative quantitative results in panel A represents the mean ± SEM of three independent experiments. *, $p < 0.05$. (C and D) Depletion of BRD7 lead to bypass of G2-M arrest. Control or BRD7-depleted HeLa cells were treated with CPT (10 nM) for 1 h and allowed to release for indicated time intervals. The cells were harvested and analysed by flow cytometry using anti-phospho-histone H3 (Ser 10) (pH3) antibodies. The percentage of mitotic cells positive for phospho-histone H3 (pH3) is indicated. (D) Quantitative results representing the mean ± SEM of three independent experiments. Error bars indicate SEM. *, $p <0.05$. (E and F) Control or BRD7-depleted HeLa cells were treated with CPT (10 nM) for 1 h or left untreated and subsequently grown in the presence of paclitaxel (Taxol; 2 μM) for indicated times. The pH3 positive cells were determined by flow cytometry using indicated antibodies. (F) Quantitative results of the percentage of mitotic cells representing the mean ± SEM of three independent experiments. Error bars indicate SEM. *, $p <0.05$. (G and H) BRD7 depletion display a specific defect in checkpoint maintenance. Control and BRD7-depleted HeLa cells were allowed to repair for indicated time intervals after cells exposure to CPT (10 nM, 1 h) or 2 Gy IR and cell lysates subjected to western blot analysis with indicated antibodies. n.s., not significant; **$P < 0.01$, Student's t-test.

Figure S9

A

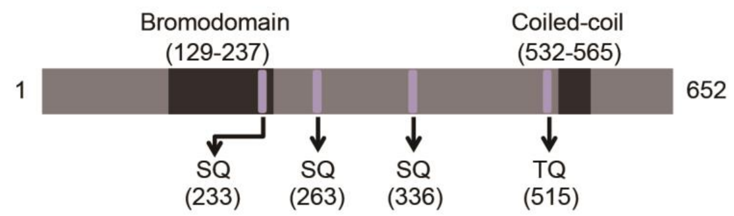

B

	Ser233	Ser263
Human	227-SGMKILSQERIQSL-240	257-KDGTDTSQSGEDGG-270
Chimpanzee	227-SGMKILSQERIQSL-240	257-KDGTDTSQSGEDGG-270
Rhesus	227-SGMKILSQERIQSL-240	257-KDRTDTSQSGEDGG-270
Dog	237-SGMKILSQERIQSL-250	267-KDRTDTSQSGEDSG-280
Mouse	227-SGMKILSQERIQSL-240	257-KERTDACQSGEDSG-270
Rat	227-SGMKILSQERIQSL-240	257-KDRADACQGGEDSG-270

	Ser336	Thr515
Human	330-TRRLVNSQCEFERR-342	509-GRLDSSTQDRLIAL-522
Chimpanzee	330-TRRLVNSQCEFERR-342	508-GRLDSSTQDRLIAL-521
Rhesus	330-TRRLVNSQCEFERR-342	509-GHLDSSTQDRLIAL-522
Dog	340-TRRLVNSQCEFERR-353	518-GRLDSSNQDRLTAL-531
Mouse	330-TRRLANSQCEFERR-342	508-GRLESSSQDRLTAL-521
Rat	330-TRRLANCQCEFERR-342	509-GRLESSGQDRLTAL-573

C

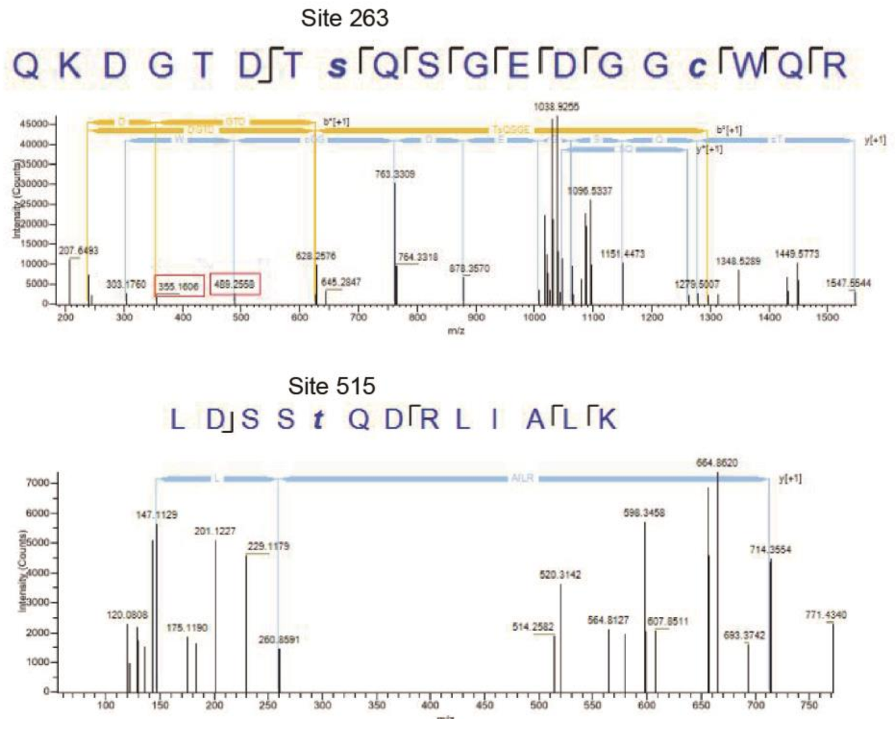

Figure S9. Identification of BRD7 phosphorylation at Ser 263 and Thr 515 through

mass spectrometry. (A) Schematic presentation of potential phosphorylation SQ/TQ motifs in BRD7. (B) Alignment of candidate phosphorylation sites in BRD7 from different species. (C) BRD7 phosphorylation was analysed by mass spectrometry. SFB-BRD7 were overexpressed in HEK293T cells, purified by S beads and isolated through SDS-PAGE. The SFB-BRD7 band was harvested and subjected to ESI-QUAD-TOF mass spectrometry.

Figure S10

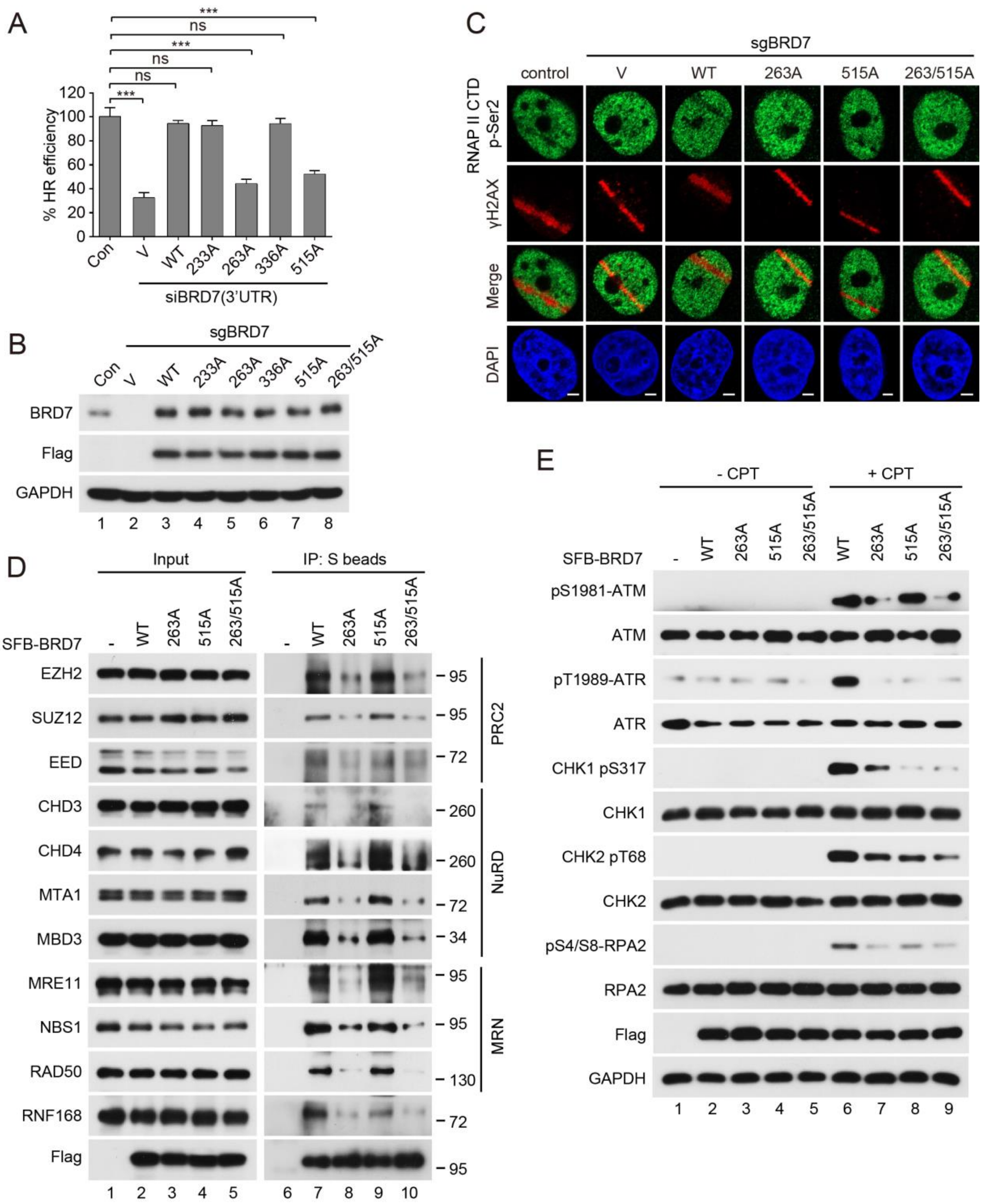

Figure S10. Phosphorylation of BRD7 at Ser 263 by ATM is required for the recruitment of PRC2, NuRD complexes and RNF168 to DSBs sites and induction of transcriptional repression, activation of ATM/ATR signalling pathway.

(A) Both BRD7 Ser 263 and Thr 515 phosphorylation are required for HR repair. BRD7-knockdown cells (HeLa DR-GFP) were reconstituted with BRD7 wild-type and indicated mutants and subjected to HR repair analysis as previously described. Quantification results are the average of three independent experiments and are shown as mean ± SEM. (B) Endogenous BRD7 is depleted by CRISPR/Cas9 approach and substituted with indicated Flag-BRD7 in HeLa cells. The stable cells were lysed with RIPA buffer, and lysates were subjected to western blot. (C) Exclusion of phosphorylation of RNAP II on Ser 2 to laser damage were reduced in S263A mutant and double-mutant S263A/T515A of BRD7. Wild-type and indicated mutants BRD7 were transfected into HeLa cells depleted of endogenous BRD7 and subjected to UV laser, and endogenous RNAP II CTD p-Ser 2 accumulation was analysed by immunofluorescence. Representative images after DNA damage are shown. Scale bars, 2 μm. (D) BRD7 Ser 263 phosphorylation is required for the association with PRC2, NuRD and MRN complexes, and ubiquitin E3 ligase RNF168. HeLa cells stably expressing SFB-BRD7 wild-type and indicated mutants were treated with CPT (1 μM, 1 h) and lysed with RIPA buffer, followed by IP using S beads, and Western blot was performed with indicated antibodies. (E) Ser 263 phosphorylation of BRD7 is required for activation of both ATM/ATR signalling, while Thr 515 phosphorylation only contributes to ATR signalling pathway. Wild-type and indicated mutants SFB-BRD7 were transfected into HeLa cells depleted of endogenous BRD7 and cells were lysed with RIPA buffer after treatment with CPT (1 μM, 1 h), followed by western blot analysis with indicated antibodies. n.s., not significant; $^{**}P < 0.01$, Student's t-test.

Table S1. siRNA Sequences

siRNA	Source	Sequence (5' – 3')
ATM	GenePharma	GGGCAAUAUUUCAAAUUAA
ATR	GenePharma	GAACAACACUGCUGGUUUG
DNA-PK	GenePharma	GATCGCACCTTACTCTGTT
BAF47#1	GenePharma	GGACATGTCAGAGAAGGAGAAC
BAF47#2	GenePharma	GGAGAACTCACCAGAGAAGTT
BAF57#1	GenePharma	AAGGAGAACCGTACATGAGCA
BAF57#2	GenePharma	GGAACCAGTGATAGTAACA
ARID1A#1	GenePharma	TAAATAGCTGTGTCTCGCT
ARID1A#2	GenePharma	GCCTGATCTATCTGGTTCAAT
ARID1A#3	GenePharma	CCGTTGATGAACTCATTGGTT
PBRM1#1	GenePharma	GCACTCAGCTATACCACAA
PBRM1#2	GenePharma	GTTAGAAGGTGGAGATGAT
PBRM1#3	GenePharma	GATATGAGCTGTATAAGAA
ARID2#1	GenePharma	AGCTCCAATTCCTTGTGAAGT
ARID2#2	GenePharma	ATGTAGGAAATGGTGAGATAT
BRD7#1	GenePharma	GUCCCUCAUACAGAGAAAU
BRD7#2	GenePharma	GACUCGUGAGGAAGGAAUG
BRD9#1	GenePharma	GGAGAAGCCTCTAAAGCTAGT
BRD9#2	GenePharma	GTCCTATCCCGACGTTTCTGT
GLTSCR1#1	GenePharma	CCCAGGCCATGCTCAATAAAT
GLTSCR1#2	GenePharma	GATGGTAATGATCGACCGAAT
CHD4#1	GenePharma	CCCAGAAGAGGATTTGTCA
CHD4#2	GenePharma	CAGTTACCAAGAAGACTTA
LSD1	GenePharma	GCCTAGACATTAAACTGAATA
RNF168#1	GenePharma	GACACTTTCTCCACAGATA
RNF168#2	GenePharma	CAAAGTAAGGCCTGGTAAA
RAD51	GenePharma	CUAAUCAGGUGGUAGCUCA
CtIP	GenePharma	GCUAAAACAGGAACGAAUC
NBS1	GenePharma	GGAAGAAACGTGAACTCAA
MBD2#1	GenePharma	GCGAAACGATCCTCTCAAT
MBD2#2	GenePharma	GAAAGATGATGCCTAGTAA
MBD2#3	GenePharma	GCAAGAGCGATGTCTACTA
MBD3#1	GenePharma	TGACCAAGATTACCAACCA
MBD3#2	GenePharma	GAACGCCTTCGACATTGCT
MBD3#3	GenePharma	CCCAGCAACAAGGTCAAGA
BRG1	GenePharma	CTCTCTCAACGCTGTCCAA

Table S2. Primary Antibodies

Antibody	Source	Identifier	Dilution	Specificity
GFP	Cell Signlling	#2956	1:1000	WB
RNAP Ⅱ	Bethyl	A300-653A-M	1:200	IF
RNAP Ⅱ CTD p-Ser2	Bethyl	A300-654A-M	1:200	IF
RNAP Ⅱ CTD p-Ser5	Bethyl	A304-408A-M	1:200	IF
RNAP Ⅱ CTD p-Ser7	Cell Signlling	#13780	1:200	IF
H2A-K119Ub	Millipore	05-678	1:200	IF / ChIP
H2AX (pS139)	BD Pharmingen	560446	1:200	IF
H2AX (pS139)	Signalway Antibody	#11268	1:1000	WB
BAF47	BD Pharmingen	612110	1:1000	WB
BAF57	ABclonal	A5361	1:1000	WB
ARID1A	Bethyl	A301-040A-M	1:1000	WB
BAF180	Bethyl	A301-591A-M	1:1000	WB
BRD7	Bethyl	A302-304A-M	1:1000	WB / ChIP / IP
ARID2	ABclonal	A8601	1:1000	WB
BRD9	Bethyl	A303-781A-M	1:1000	WB
GLTSCR1(H-10)	Santa Cruz	SC-515086	1:500	WB
CHD4	Cell Signlling	#11912	1:1000	WB
LSD1	Bethyl	A300-215A-M	1:2000	WB
RAD51	Cell Signlling	#8875	1:1000	WB / ChIP
RPA2	Cell Signlling	#2208	1:1000	WB
RPA2	Origene	TA500786	1:200	IF
Phospho-RPA2	Bethyl	A300-245A-M	1:1000	WB
Tubulin (F-1)	Santa Cruz	SC-166729	1:1000	WB
Histone H3	Cell Signlling	#9717	1:2000	WB
Cyclin A2	Cell Signlling	#4656	1:1000	WB
Cyclin E2	Cell Signlling	#4132	1:1000	WB
EZH2	BD Pharmingen	612666	1:1000<br>1:200	WB<br>IF
EZH2	ABclonal	A13867		IP

EED	ABclonal	A12773	1:1000	WB
SUZ12	ABclonal	A7786	1:1000	WB / IP
BMI1	Cell Signlling	#6964	1:1000<br>1:200	WB<br>IF
RING1A	Cell Signlling	#13069	1:1000	WB
RING1B	Cell Signlling	#5694	1:1000	WB
CHD3	Cell Signlling	#4241	1:1000	WB
HDAC1	Cell Signlling	#5356	1:1000	WB
HDAC2	Cell Signlling	#5113	1:1000	WB
MBD3	Cell Signlling	#14540	1:1000	WB
MTA1	Cell Signlling	#5647	1:1000	WB
RBAP46	Cell Signlling	#6882	1:1000	WB
RNF168	ABclonal	A3556	1:1000	WB / IP
RNF168	Millipore	#ABE367	1:200	IF
RNF8	Signalway Antibody	#21699	1:1000	WB
H3K27me3	Cell Signlling	#9733		ChIP
Flag	Proteintech	20543-1-AP	1:1000	WB
BRCA1	Santa Cruz	SC-6954	1:200	IF
53BP1	BD Pharmingen	612523	1:200	IF
Chk1	Cell Signlling	#2360	1:1000	WB
pSer317-Chk1	Cell Signlling	#12302	1:1000	WB
pSer296-Chk1	Cell Signlling	#2349	1:1000	WB
Chk2	Cell Signlling	#6334	1:1000	WB
pThr68-Chk2	Cell Signlling	#2661	1:1000	WB
NBS1	Cell Signlling	#14956	1:1000	WB
ATM	Bethyl	A300-299A-M	1:1000	WB / IP
pSer1981-ATM	Cell Signlling	#5883	1:1000	WB
(pS/pT)Q	Cell Signlling	#6966	1:1000	WB / IP
Phospho-Histone H3(Ser10)	Cell Signlling	#3465	1:200	FACS
ATR	Bethyl	A300-137A-M	1:1000	WB / IP
MBD2	Proteintech	55200-1-AP	1:1000	WB

MBD3	Proteintech	14258-1-AP	1:1000<br>1:200	WB<br>IF
Ku70	ABclonal	A7330	1:200	IF
Ku80	Cell Signlling	#2180	1:200	IF
RNF8	Proteintech	14112-1-AP	1:200	IF
